# *Salvia* Species as Sources of Natural Products with Antiprotozoal Activity

**DOI:** 10.3390/ijms19010264

**Published:** 2018-01-16

**Authors:** Núria Llurba-Montesino, Thomas J. Schmidt

**Affiliations:** Institut für Pharmazeutische Biologie und Phytochemie (IPBP), University of Münster, PharmaCampus, Corrensstraße 48, D-48149 Münster, Germany; n_llur01@uni-muenster.de

**Keywords:** *Salvia*, natural products, neglected tropical diseases (NTDs), *Plasmodium*, *Leishmania*, *Trypanosoma*, global health

## Abstract

Natural products from plants have been used since ancestral times to treat a wide variety of diseases worldwide. Plants of the genus *Salvia* (Sage) have been reported to be used for the prevention and treatment of various diseases and ailments. In particular, some *Salvia* species have been used in traditional medicine to treat diseases caused by protozoan parasites of the genera *Trypanosoma*, *Leishmania* and *Plasmodium* and scientific studies have demonstrated the activity of various isolated constituents from these plants against these pathogens. The current review attempts to give a critical overview of published information about the antiprotozoal activity of species of the genus *Salvia* and their chemical constituents. It is meant to give a unified overview of these results in order to avoid repetitions caused, e.g., by limited access to some primary reports, and to stimulate further research to possibly facilitate the development of new molecular leads against protozoal neglected tropical diseases (NTDs) based on *Salvia* constituents.

## 1. Introduction

*Salvia* (Sage) is the largest genus of the family Lamiaceae. It is widespread in various regions around the world comprising the Mediterranean area, South Africa, Central and South America as well as Asia [1,2], and some of its representatives are also cultivated and exported to other regions worldwide. The genus comprises around 1000 species [2,3], many of which have long been used as flavoring agents, aromatics, and ornamental plants as well as in perfumery and cosmetics. *Salvia* species are also known for a wide variety of medicinal uses in folk medicine. In fact, the name *Salvia* is derived from the Latin word salvare, which refers to its healing qualities. In Europe, the leaves of *Salvia officinalis* L. and *Salvia triloba* L. (a synonym of *S. fruticosa* Mill.) are extensively used as a mouth wash against inflammations in the oral cavity [1]. The roots of *Salvia miltiorrhiz*a Bunge, a species endemic to China and Japan, have long been used in Traditional Chinese Medicine (TCM) to treat circulation-related diseases [4]. *Salvia sclarea* L. is used as a remedy to alleviate night sweats associated with menopause or tuberculosis [1] and also used in perfumery as a flavoring [5]. South African species such as *Salvia stenophylla* Burch. ex Benth., *Salvia runcinata* L. and *Salvia repens* Burch. ex Benth. have long been used as disinfectants and as purgative [6].

Infectious diseases caused by unicellular eukaryotic parasites (“protozoans”) of the genera *Trypanosoma* (Chagas disease and sleeping sickness), *Leishmania* (different forms of leishmaniasis)—which are among the list of 20 neglected tropical diseases (NTDs) recognized by the WHO—and *Plasmodium* (malaria), threaten the life of millions of people worldwide. The majority of affected populations are from tropical middle and low income countries, but since parasites do not respect national and political borders, protozoal infections threaten other populations and thus constitute a global health problem. Both the lack of safe and available treatments, the threatening increase of drug-resistance against current therapies and the insecticide resistance for vector control make the research on and development of new anti-protozoal drugs an urgent need.

Each of the mentioned parasites has a complex life cycle in which various forms occur subsequently in different host organisms (insect vectors, infected mammalians; for a brief overview see, e.g., [7], or specialized parasitological literature). These life forms differ from each other in physiology and, hence, drug susceptibility. It is hence of great importance when searching for new active entities to use in vitro bioassay models of the clinically most relevant forms of the parasites. These are trypomastigote forms as present in the bloodstream and cerebroventricular fluid in case of *T. brucei*, amastigote forms as present in host cells in case of *T. cruzi* and *Leishmania* species and intraerythrocytic forms in case of *Plasmodium* spp. (for an overview on assay systems, see, e.g., [7]).

*Salvia* is distributed worldwide and cultivated so that some species grow where protozoal neglected diseases are endemic. Despite the large spectrum of medicinal uses of Sage species, which mainly describe them against conditions associated with fever and perspiration [6], relatively few references have reported on their traditional use against infectious diseases caused by protozoans. However, a few publications recognize their ethnomedicinal use as antimalarial, antitrypanosomal and antileishmanial remedies [6,8].

Terpenoids (essential oils as well as di- and tri-terpenes) and phenolic compounds, such as flavonoids and caffeic acid derivatives, are known to be present in aerial parts of *Salvia* species. Hundreds of secondary metabolites have been isolated from different species of *Salvia* and have been tested for a wide range of biological activities [5].

Different research groups have been investigating *Salvia* species as well as some of their isolated constituents, reporting them as possible antiprotozoal agents against parasites of the genera *Plasmodium*, *Trypanosoma* and *Leishmania*. Even though some of the reported chemical constituents showed promising activities and encouraging results, to the best of our knowledge, no data on research focused on a direct applicability or further development of *Salvia* or its constituents (e.g., on mechanism of action studies, structural optimization or clinical trials against protozoal infections) exist.

This review is intended to summarize and give a critical overview of the published information concerning the antiprotozoal activity of *Salvia* species and their isolated compounds. This collection of data is expected to help avoid research repetitions due, e.g., to limited access to primary data sources, and, possibly, to facilitate the development of new molecular leads against protozoal NTDs based on *Salvia* constituents.

The following specific abbreviations are used throughout this review: Neglected Tropical Diseases (NTDs), *Trypanosoma brucei rhodesiense* (*Tbr*), *Trypanosoma brucei gambiense* (*Tbg*), *Trypanosoma brucei brucei* (*Tbb*), *Trypanosoma cruzi* (*Tc*), *Plasmodium falciparum* (*Pf*), *Leishmania donovani* (*Ld*), *Leishmania major* (*Lm*), *Leishmania infantum* (*Li*), *Leishmania killicki* (*Lk*), *Leishmania guyanensis* (*Lg*), *Leishmania tropica* (*Lt*), Chagas disease (CD), sleeping sickness or Human African Trypanosomiasis (HAT), concentration causing half-maximal growth inhibition (IC_50_), and selectivity index (SI = IC_50_ (mammalian control cells)/IC_50_ (parasite)).

## 2. Methodology

An online literature search for information available about antiprotozoal activity of *Salvia* species and their constituents was conducted, based on common database sources (Web of Science, SciFinder, PubMed-NCBI and Google Scholar). In this search, combinations of the keyword *Salvia* with the terms antiprotozoal, antitrypanosomal, antimalarial, antileishmanial, malaria, Chagas disease, sleeping sickness, African trypanosomiasis, *Leishmania*, *Trypanosoma* and *Plasmodium* were applied.

**Figure 1:**
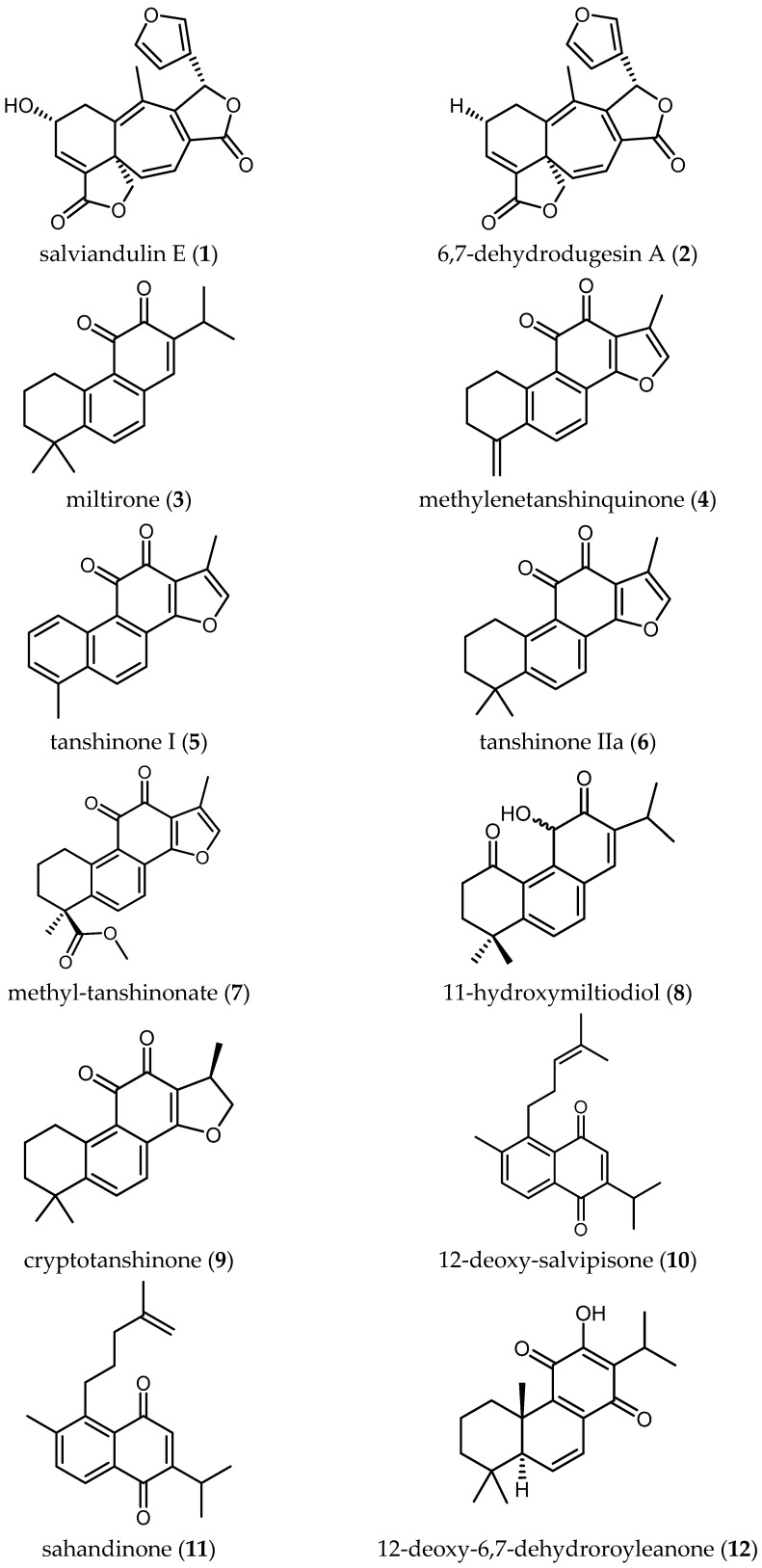

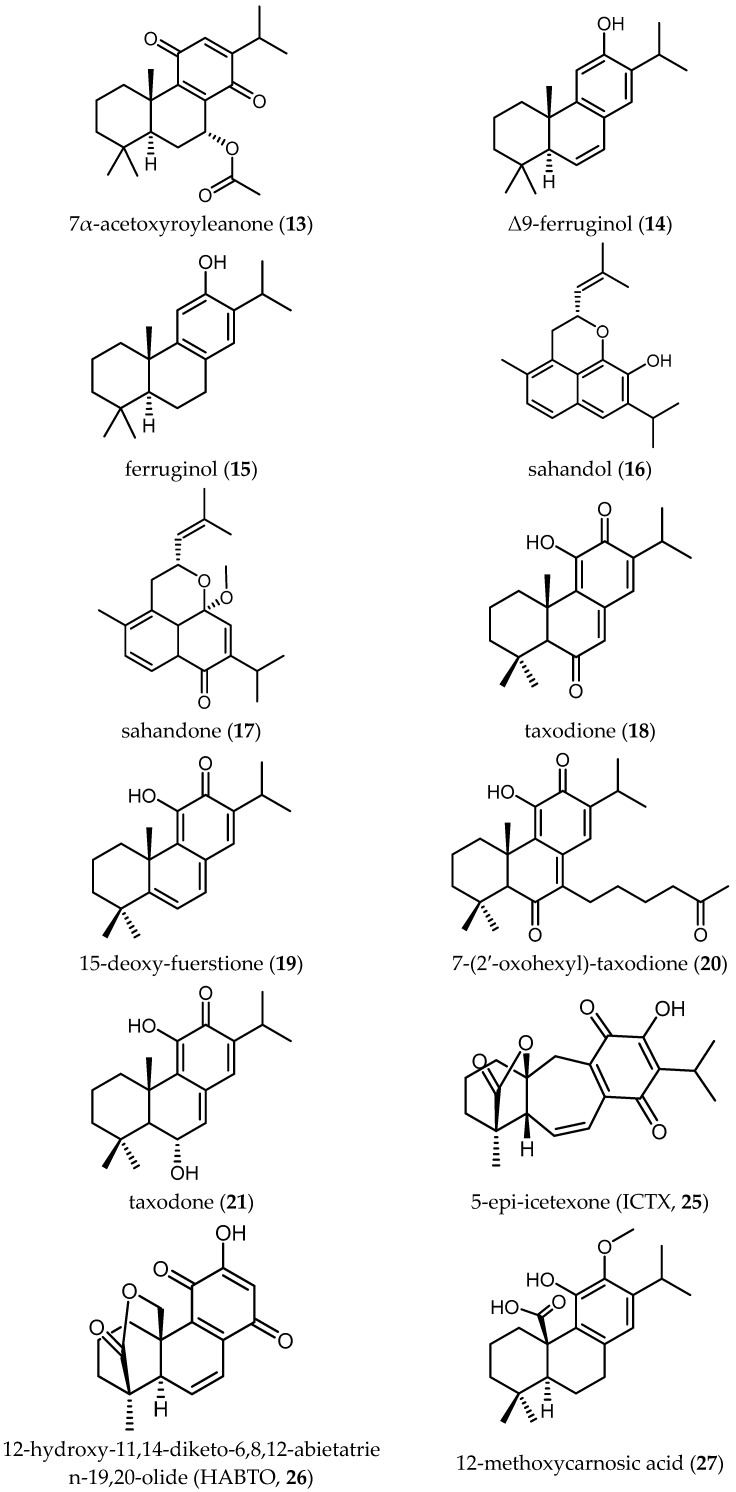

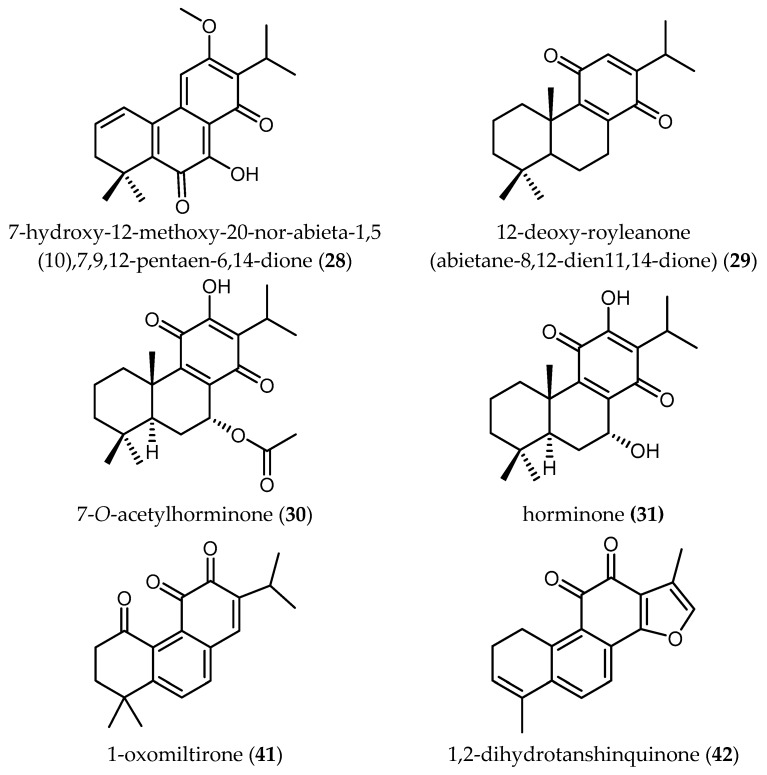
Diterpenoids of *Salvia* with antiprotozoal activity.

**Figure 2:**
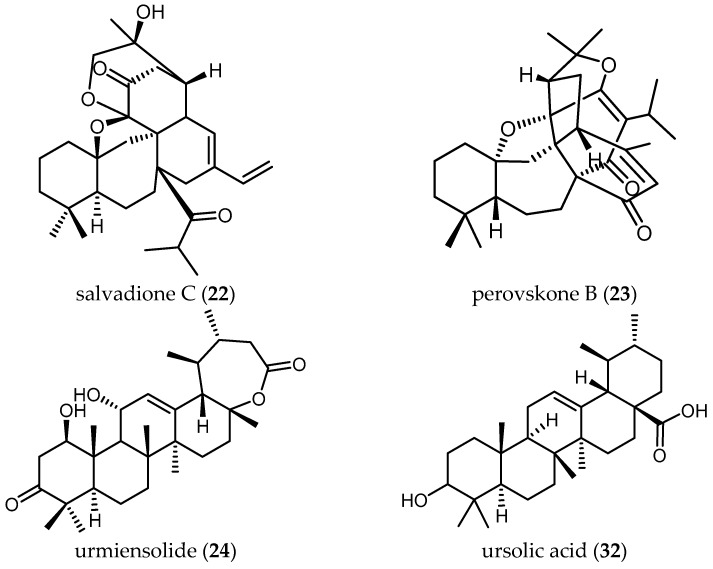

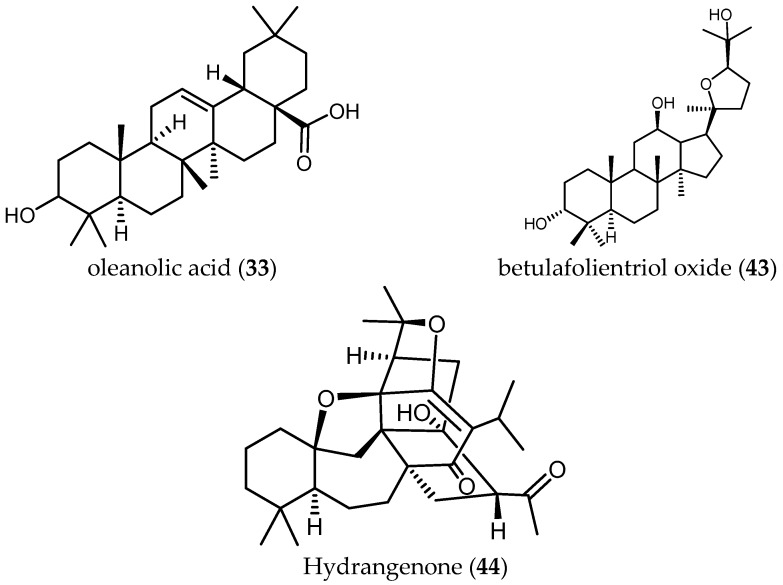
Triterpenes and related terpenoids of *Salvia* with antiprotozoal activity.

**Figure 3:**
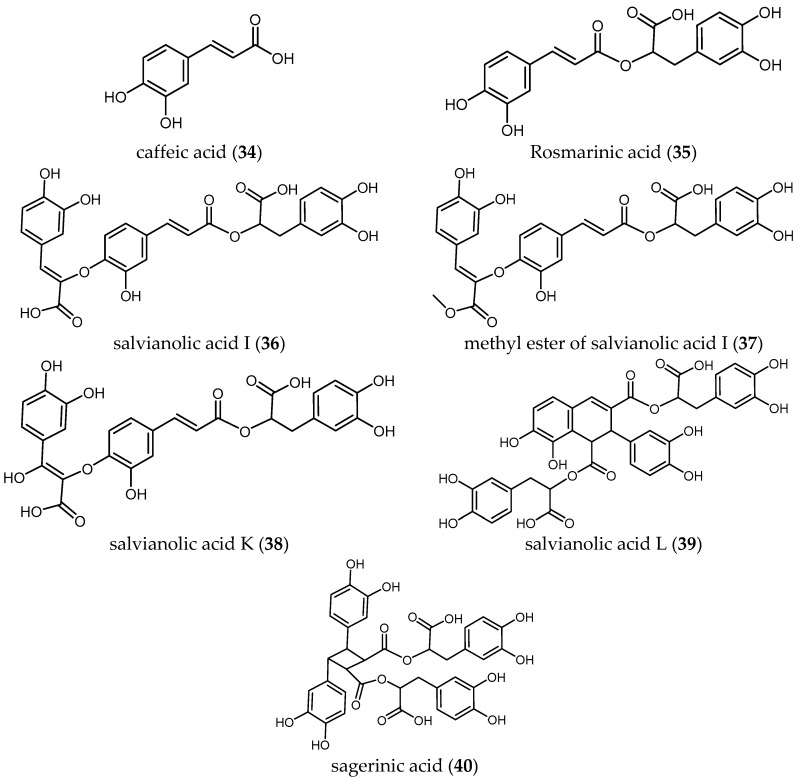
Phenolic compounds of *Salvia* with antiprotozoal activity.

**Figure 4:**
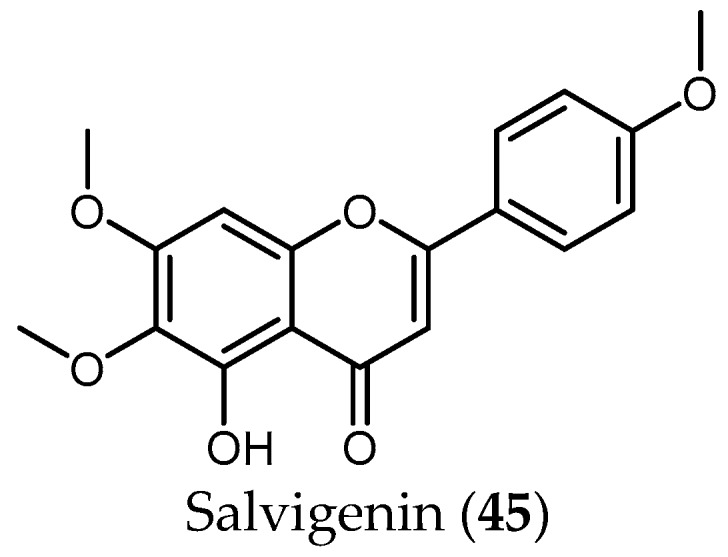
Flavonoids of *Salvia* with antiprotozoal activity.

A total number of 34 publications from the years 2002 to 2017 were obtained. The results of these studies have been ordered in this review by biological activity (i.e., the parasite against which the studies were performed) and then by structurally/biosynthetically related secondary metabolites of *Salvia* species with their antiprotozoal activity. Table 1, Table 2, Table 3, Table 4, Table 5, Table 6, Table 7 and Table 8 and Figure 1, Figure 2, Figure 3 and Figure 4 summarize the content of this review.

Thirty-five species of *Salvia* and 45 compounds isolated from *Salvia* species have been reported to be tested against at least one of the protozoans treated in this review.

## 3. Biological Activity

### 3.1. Antitrypanosomal Activity

Protozoans of the genus *Trypanosoma* cause fatal diseases which threaten the lives of millions of people worldwide.

*Trypanosoma brucei rhodesiense* (*Tbr*) and *T. brucei gambiense* (*Tbg*) are parasites transmitted by tsetse flies (*Glossina* spp.) and are etiological agents of different forms of Human African Trypanosomiasis (HAT), also known as “sleeping sickness”. HAT is endemic in 36 sub-Saharan countries [9] and its global burden was estimated to be 0.3 million disability-adjusted life years (DALYs) in 2015 [10]. Other species of *Trypanosoma* such as *T. brucei brucei* (*Tbb*) are known to be responsible for diseases in animals. The latter, together with other species such as *T. congolense*, is a causative agent of the cattle disease Nagana, also responsible for a severe economic and health problem in some parts of Africa [11]. 

The currently available diagnostic tests and treatment of the HAT require skilled personnel and, in most cases, hospitalization, to which most of the affected people living in rural areas do not have access. Moreover, the current therapies are painful and present severe side effects. All in all, new safe, orally applicable and inexpensive treatments for HAT are urgently needed [11]. The very recent positive outcome of phase III clinical trials with the synthetic compound fexinidazole [12], the first orally applicable drug against *Tbg* infection, may be seen as an encouraging step in this direction.

The American trypanosomiasis, also known as Chagas disease, is caused by the protozoan *Trypanosoma cruzi*, which is transmitted mainly by triatomine bugs (*Triatoma* spp., *Rhodnius* spp. and *Panstrongylus* spp.) but also by congenital transmission, blood transfusion or organ transplants. A number of cases of infection after ingestion of the parasites with food or drink have also been reported [13]. It is endemic in 21 countries of South America where the vectors live. Moreover, migration and travelers extended the disease around the world. In non-endemic countries such as Spain or Canada, around 50,000 and 1800 of people, respectively, are estimated to be infected with *Tc* [14]. Because of its unspecific symptomatology in early phases of infection, thousands of patients remain undiagnosed. It is estimated that the disease affects around 8 million people and causes 20,000 deaths every year [15]. The known resistance and lack of sensitivity of this parasite to existing therapies (as well as in-research-drugs) make the research in this field particularly difficult and challenging.

#### 3.1.1. Activity of Crude Extracts and Isolated Constituents from *Salvia* Species against *Trypanosma Brucei*

##### Extracts and Essential Oils

Some *Salvi*a species and compounds isolated from them have been reported to show biological activity against the etiological agents of HAT and of the cattle disease, Nagana. The reported activity data for 12 extracts obtained from three *Salvia* species against *T. brucei* spp. are summarized in Table 1. Methanol extracts and their partitions with n-hexane, chloroform and water of *Salvia tomentosa* Mill., *Salvia sclarea* L. and *Salvia dichroantha* Stapf. were tested against *Tbr* STIB 900 with IC_50_ values ranging from 1.24 to 10.96 µg/mL (named as **a1**–**c4** in Table 1), being the n-hexane partition of *S. tomentosa* Mill. (**a2**) the most active, with an IC_50_ = 1.24 µg/mL and significant selectivity with an SI = 21.16, compared with L6 rat skeletal myoblast cells [16]. More and deeper investigations with the most active extracts of these species should be performed to find the compound(s) responsible for their biological activity.

Farimani and coworkers reported on the activity of an n-hexane extract of *Salvia hydrangea* DC. ex Benth. (IC_50_ = 18 µg/mL against *Tbr* STIB 900 strain) (**d**) (Table 1) [17]. Furthermore, a dichloromethane extract from the roots of *S. miltiorrhiza* Bunge (named as **e** in Table 1) showed 97% of growth inhibition against *Tbr* at 0.81 µg/mL [4]. Moreover, a methanol-dichloromethane extract of *S. repens* Burch. ex Benth. (**f**) (Table 1) showed less promising activity with an IC_50_ value of 10.8 µg/mL [18]. 

An ethanolic extract of *Salvia spathacea* Greene. (**g**) (Table 1) was tested by Jain and coworkers against *Tbb* showing interesting IC_50_ values of 1.13 ± 0.78 µg/mL [19].

Ihsan et al. tested the essential oil of *Salvia lavandulifolia* Vahl. (**h**) (Table 1) against the trypomastigote form of *Tbb* which did not show inhibitory effects at concentration up to 20 µg/mL [20].

Recently, as part of the present authors’ own work, an extract obtained by evaporation of a commercial tincture of *S. officinalis* L. (**i**) (Table 1) was found to exhibit quite significant activity against *Tbr* with an IC_50_ of 1.86 µg/mL and an SI (determined with L6 rat skeletal myoblasts) of about 17 [21]. Isolation and characterization of the main active principles of this extract is in progress.

In most of the cases where active constituents were characterized, diterpenoids and triterpenoids were responsible for the reported antitrypanosomal activity (summarized in Table 2).

##### Diterpenoids

Salviandulin E (**1**)—a neoclerodane diterpene isolated originally from *Salvia leucantha* Cav.—and a set of synthetic analogs were tested in vitro against *Tbb* GUTat 3.1 by Aoyagi and coworkers. Compound **1** itself showed potent biological activity (IC_50_ = 2.04 µM). A similar diterpene isolated from the same plant, 6,7-dehydrodugesin A (**2**), did not show antitrypanosomal activity (IC_50_ > 37.19 µM) [22]. The synthetic derivative butanoyl 3,4-dihydro salviandulin showed the best IC_50_ = 0.14 µM and SI = 1236 [22]. Although is known that *Tbb* and *Tbr* are genetically almost identical [7], some compounds showed different bioactivity against the two parasites [7]. Therefore, studies against *Tbr* or *Tbg* should be carried out with these compounds to evaluate the biological activity against human pathogenic parasites. The improvement of the activity and selectivity of the compounds by a semi-synthetical approach are a nice example that relatively simple chemical modification of natural products can be an important step in drug development.

Some tanshinone-type diterpenoids isolated from *S. miltiorrhiza* Bunge [4] showed activity ranging from 0.5 to 26.2 µM and SI from 0.2 to 24.2. Miltirone (**3**) and methylenetanshinquinone (**4**) displayed the highest activity, both with IC_50_ values of 0.5 µM and SI of 2.6 and 24.2, respectively. Tanshinone I (**5**) also yielded a promising IC_50_ of 1.3 µM and an SI of 9.5. Less active diterpenes (**6**–**9**) isolated in the same study are also included in Table 2.

Ebrahimi and colleagues reported on four diterpene-quinones (**10**–**13**) and four abietane diterpenes (**14**–**17**) (Table 2), isolated from *Salvia sahendica* Boiss & Buhse, to have antitrypanosomal activity [23]. They exhibited interesting IC_50_ values from 1.8 to 28.1 µM against *Tbr* STIB 900 strain. Unfortunately, the low SI values (0.1–1.2) indicate the nonselective toxicity of these compounds. Chemical structural modifications of the reported compounds, as demonstrated in the case of Compound **1**, might be attempted to decrease their toxicity.

Kuźma and coworkers isolated four quinone methide-type diterpenes from the roots of *Salvia austriaca* Jacq. (**18**–**21** in Table 2) and tested them against trypomastigotes of *Tbr* STIB 900. They yielded promising IC_50_ values from 0.05 to 194.7 µM and SI from 0.69 to 38, taxodione (**18**) being the most active and promising compound, which showed IC_50_ of 0.05 µM and a favorable SI of 38 [24].

##### Triterpenoids

The triterpenoids salvadione C (**22**) and perovskone B (**23**) isolated from *S. hydrangea* DC. ex Benth. displayed low activity against *Tbr* STIB 900, with IC_50_ values of 4.33 and 15.92 μM, respectively [25].

Farimani also reported one triterpenoid with an ε-lactone E-Ring showing biological activity against *Tbr* STIB 900 strain. Urmiensolide (**24**), isolated from *Salvia urmiensis* Bunge—an endemic *Salvia* species in the West Azerbaijan province of northwestern Iran—showed in vitro antitrypanosomal activity with an IC_50_ value of 5.6 μM and SI of 33 [17].

Further research on the mechanism(s) of action accompanied by studies on in vivo efficacy, bioavailability and possible metabolic routes would appear necessary to evaluate whether such triterpenes have a potential for further drug development.

#### 3.1.2. Activity of Crude Extracts and Isolated Constituents from *Salvia* Species against *Trypanosoma Cruzi*

The antitrypanosomal activity of *Salvia* species against the etiological agent of Chagas disease (CD) has been less studied in comparison with the other protozoan diseases. However, some promising results have been found in this field (Table 3 and Table 4).

##### Extracts

In the same study cited above, with respect to *Tbr* [16], the methanol extracts and their partitions with n-hexane, chloroform and water obtained from *S. tomentosa* Mill., *S. sclarea* L. and *S. dichroantha* Stapf. (named as **a1**–**c4** in Table 3) were tested against *Tc* trypomastigotes. In contrast with the relatively promising biological activity values against *Tbr*, the IC_50_ values against *Tc* were considerably higher, ranging from 18.17 µg/mL to >90 µg/mL and displayed unfavorable SI values ranging from 0.89 to ≥2.15 [16].

Furthermore, a dichloromethane-methanol (1:1) extract of *S. repens* Burch. ex Benth. (named as **f** in Table 3) was tested against *Tc* trypomastigotes showing unfavorable antitrypanosomal activity with IC_50_ = 36.2 µg/mL and SI = 1.15 [26].

##### Diterpenoids

A novel icetexane diterpene, 5-epi-icetexone (ICTX) (**25**), isolated from aerial parts of *Salvia gilliessi* Benth.—a *Salvia* species native to an endemic Chagas region—was tested against cultured epimastigotes of *Tc* (Tulahuen strain) to investigate the influence on proliferation, viability and morphology of the parasites and understand the mechanism of action. Compound **25** showed an antiproliferative effect on the *Tc* epimastigotes beginning at a concentration of 2.8 µM and its IC_50_ was determined as 6.5 ± 0.75 µM. The compound exhibited low cytotoxicity to mammalian cells. A comparison with other strains (CL-Brener, Dm28c) was also carried out but no significant differences were observed. An interaction with the DNA of the parasite has been suggested as one the mechanisms of action responsible for the cytostatic effect, but it was concluded that multiple mechanisms should be involved [27]. Further investigation with other parasite stages was carried out by Lozano and colleagues testing **25** against amastigotes with observable effects at concentrations of 1.5–3.8 μM. However, no IC_50_ values have been reported [28]. The promising activity of **25** against *Tc* justified the further studies in vivo in Swiss albino mice infected with *Tc* (Tulahuen strain *Tc* VI). The survival of animals treated daily with 10 mg/kg/day of **25** increased significantly in comparison with the control (saline or 0.1% DMSO), and also a weekly decrease of parasitemia was observed. Moreover, the treatment with **25** prevented the nest formation of amastigotes within muscle fibers [29]. All these data make Compound **25** a favorable lead compound to be considered for further drug development steps.

The effect of the abietane diterpene 12-hydroxy-11,14-diketo-6,8,12-abietatrien-19,20-olide (HABTO) (**26**) isolated from the aerial parts of *Salvia cuspidata* subsp. *gilliesii* (Benth.) J.R.I.Wood on *Tc* epimastigotes was also studied by Lozano and colleagues [30]. This compound was reported to inhibit parasite growth at moderate concentrations (IC_50_ ≈ 16.6 µM) and described to be of low toxicity for mammalian cells (SI value not available). In this study, some chemical modifications rendering the compound more lipophilic also succeeded to improve the trypanocidal activity. The 12-trimethylsilyloxy derivative of **26** showed the highest toxicity to the parasite, representing another example that small chemical modifications can already improve the activity of the native natural products.

In the same study cited above with respect to *Tbr*, Kuźma and coworkers tested four diterpenes with a quinone moiety isolated from the roots of *S. austriaca* Jaqc. (**18**–**21**) against amastigotes of *Tc* (Tulahuen strain C2C4 containing the β-galactosidase Lac Z) [24]. The IC_50_ ranged from 7.11 µM to 146.9 µM, taxodione (**18**) being again the most active compound. Unfortunately, the unfavorable SI values (from 0.27 to 0.91) indicated non-selective toxicity against mammalian cells.

### 3.2. Antileishmanial Activity

Leishmaniasis is a group of diseases caused by more than 20 protozoan species of the genus *Leishmania*, transmitted by the bite of infected sandflies (genera *Phlebotomus* in the Old World, *Lutzomyia* in the New World). From the different manifested forms of this disease, cutaneous leishmaniasis is the most common and the visceral leishmaniasis—also known as Kala-Azar—is the most serious form, leading to death if untreated. According to estimates by WHO, 1.3 million new cases occur every year, and it is estimated that 20,000–30,000 people die annually of visceral leishmaniasis [31].

Notwithstanding the positive breakthrough in terms of diagnostic programs and the reduction of prices for the treatment, the mortality and morbidity due to leishmaniasis worldwide is still worrying [32]. The lack of vaccines against *Leishmania* spp. and the increasing resistance of the parasites against current therapies, make research and development of new treatments urgent.

Natural products from plant sources have long been studied as potential antileishmanial leads [33]. However, none of the investigated compounds seem to have reached clinical investigation so far.

In the case of *Salvia*, infusions from the flowers have reportedly been used in Iranian folk medicine as antileishmanial treatment [8]. Sixty-one species of *Salvia* occur in Iran, seventeen of which are endemic [17]. No further investigations, however, have been carried out to prove the efficacy of this treatment, but some diterpenes, triterpenes and extracts of other *Salvia* species have been reported in the published literature to be active against different species of *Leishmania* (Table 5 and Table 6).

#### 3.2.1. Extracts and Essential Oils

Several research groups investigated the antileishmanial activity of different species of *Salvia*. The above-mentioned extracts of different polarity from three *Salvia* species (*S. tomentosa* Mill., *S. sclarea* L. and *S. dichroantha* Stapf.) were also tested against axenic amastigotes of *Ld* strain MHOM/ET/67/L82, showing IC_50_ values ranging from 1.81 to >90 µg/mL and favorable SI values ranging from 7.63 to 29.47 (Table 5). The most active extract in this case was the chloroform partition of *S. tomentosa* Mill. (named as **a3** in Table 5) which displayed an IC_50_ of 1.81 µg/mL and an SI value of 29.47 [16].

In the same study cited above with respect to *Tc* [26], the dichloromethane-methanol (1:1) extract of *S. repens* Burch. ex Benth. was tested against axenic amastigotes of *Ld.* The IC_50_ was considerably better than in *Tb*, being 5.36 µg/mL, with an SI value of 7.74.

Ihsan and coworkers tested the essential oil of *S*. *lavandulifolia* Vahl. (**h**) against different stages of *Ld.* No leishmanicidal effects were observed at concentration up to 20 µg/mL (Table 5) [20].

The essential oil of *S. officinalis* L. (**i1** in Table 5) was tested by Essid and coworkers against the promastigote forms of *Lm* and *Li*, yielding IC_50_ values of 3.40 ± 0.16 and 2.67 ± 0.33 µg/mL, and moderate SI values of 5.92 and 7.54, respectively [34]. Moreover, Nikmehr et al. tested the antiprotozoal activity of the methanolic maceration of *S. officinlais* L. (**i2** in Table 5) against amastigotes and promastigotes of *Lm*. The macerated extract exhibited an unfavorable IC_50_ value against the promastigotes of 184 ± 11.17 µg/mL. Furthermore, at this concentration it yielded 58% of lethality of parasite internalized into macrophages (cell line infected with *Lm* amastigotes) and 8% of toxicity [35].

A.R. Khan and M.J. Khan investigated the antileishmanial activity of a methanolic extract from leaves from *Salvia bucharic*a Popov. and different solvent partitions (named as **j**-**j4** in Table 5) [36]. The extract demonstrated a low IC_50_ value of 72.31 µg/mL. The following partitions (chloroform, acetone and water partitions) showed IC_50_ values ranging from 30.51 to >100 µg/mL, the aqueous partition being the most active (Table 5). More research on the polar fraction should be carried to possibly identify single compounds with higher antileishmanial activity.

Three solvent partitions of *Salvia verbenaca* (L.) Briq. ssp *verbenaca* Maire (*S. clandestina* Batt. non L.) were tested by Et-Touys et al. against promastigote forms of *Lm*, *Lt* and *Li* [37]. The methanolic partition did not exhibit antileishmanial activity against any of the tested parasites. However, the dichloromethane partition showed better IC_50_ values ranging from 24.56 to 33.77 µg/mL. The most active extract was the n-hexane partition, which showed moderate activity against *Li* (IC_50_ = 14.11 µg/mL) but lower activity against *Lm* and *Lt*, with IC_50_ of 155.43 and 148.23 µg/mL, respectively (Table 5). 

#### 3.2.2. Diterpenes

The reported diterpenes isolated from *Salvia* species displaying antileishmanial activity belong to the abietane group. Their activity seems to be higher in comparison with the activity against the other parasites reported above, since most of them showed IC_50_ lower than 1 µM (Table 6).

Mokoka and colleagues reported on 12-methoxycarnosic acid (**27**), isolated from the moderately active dichloromethane-methanol (1:1) extract of the whole plant of *S. repens* Burch. ex Benth. (IC_50_ = 5.36 µg/mL) [26], showing a low IC_50_ value of 0.75 μM against axenically cultured amastigotes of *Ld* and a favorable SI of 23.06 [18]. These promising values make this compound interesting as a lead compound. However, no further studies to fully evaluate its potential have been found.

Two new abietane diterpenes derivatives isolated from the acetone extract of the roots of *Salvia cilicica* Boiss and Kotschy, 7-hydroxy-12-methoxy-20-nor-abieta-1,5(10),7,9,12-pentaen-6,14-dione (**28**) and abieta-8,12-dien-11,14-dione (12-deoxy-royleanone) (**29**), were tested against promastigotes and intracellular amastigotes of both *Ld* and *Lm* [38]. Both diterpenes were shown to be more active against the clinically more relevant amastigote stages and displayed better antileishmanial activity against *Ld*, with IC_50_ values of 0.17 and 0.121 µM, respectively. The activity of **28** and **29** against *Lm* was lower with IC_50_ values of 0.287 and 0.18 µM, respectively. Unfortunately, the SI values did not show particularly selective toxicity against the parasites, since they ranged from 0.17 to 2.22.

Búfalo et al. isolated four diterpenes (**15**, **18**, **30** and **31** in Table 6) from the roots of *Salvia deserta* Schang.—an Asian *Salvia*—and tested their activity against promastigotes of *Ld* [39]. The IC_50_ ranged from 1.46 ± 0.52 μM to 29.43 ± 3.01 μM. Taxodione (**15**), a diterpenoid quinone methide previously reported to yield promising activity against *Tbr* (see Section Diterpenoids), was the only compound with significant activity yielding an SI of 10.38, which could also make this compound interesting to be considered a good starting point for further development of novel antileishmanials.

#### 3.2.3. Triterpenes

Tan and colleagues tested the antileishmanial activity of two triterpenes, ursolic acid (**32**) and oleanolic acid (**33**), isolated from *S. cilicica* Boiss and Kotschy [38]. They showed to be more potently active against amastigote forms of *Ld* than *Lm*, with IC_50_ values of 0.013 and 0.063 μM against *Ld* and IC_50_ values of 0.007 and 0.12 μM in the case of *Lm*. The ratio of cytotoxic over antileishmanial activity was unfavorable for both compounds with SI values ranging from 0.3 to 2.22. These ubiquitous triterpenes have been reported many times to possess anti-protozoal activity [18,29,40]. Since the level of activity and selectivity is not very high, their usefulness as anti-protozoal lead structures is questionable. Some chemical changes might be an option to improve their selectivity towards parasites.

#### 3.2.4. Phenolic Compounds

To best of our knowledge, less has been studied about the antiprotozoal activity of phenolic compounds of *Salvia* species. Radtke et al. isolated seven phenolic compounds (**34**–**40**) from *S. officinali*s L. and tested them against the amastigote and promastigote forms of several *Leishmania* species (*Ld*, *Lm*, *Lg* and *Lk*) [41]. None of the phenolic compounds showed selective activity when tested against the promastigote stages of the four *Leishmania* species (IC_50_ ranging from 0.70 to 2.4 μM) compared with the high and selective activities observed against the intracellular amastigote stages (IC_50_ values ranging from 0.004 to 0.176 μM) (Table 6).

Caffeic acid (**34**), the monomer unit of all tested phenolic compounds, was the most active compound against all tested parasites. This may indicate that the size of the molecule could be an important criterion responsible of the antileishmanial activity.

Concerning the two species of *Leishmania* with higher morbidity and mortality, the IC_50_ values of the phenolic compounds against *Lm*, an etiological agent of cutaneous leishmaniasis, ranged from 4.4 to 160.4 nM. Compound **34** exhibits the higher activity, followed by the mehtyl ester of salvianolic acid I (**37**), salvianolic acid K (**38**) and salvianolic acid L (**39**) with IC_50_ values of 0.0108, 0.0183 and 0.0203 μM, respectively. Interestingly, salvianolic acid I (**36**) showed sixteen-fold lower activity (IC_50_ = 0.1604 μM) than its methyl ester derivative (**37**), which again confirms that small modifications in molecules can represent a huge difference in terms of bioactivity. Further studies with methylated esters of **38**–**40** could be an interesting starting point for antileishmanial compounds.

Concerning the antileishmanial activity against *Ld*, etiological agent of visceral leishmaniasis, compound **34** exhibited the highest activity (IC_50_ = 0.0061 μM and favorable SI > 360), followed by salvianolic acid L (**39**) and salvianolic acid K (**38**) with IC_50_ values of 0.0154 and 0.0182 μM, respectively. Again, the methyl ester derivative (**37**), displayed significantly higher antileishmanial activity (IC_50_ 0.0185 μM) than compound (**36**) (IC_50_ = 0.1758 μM). Compound **37** also showed higher selectivity against the parasite than compound **36** (SI > 37.63 and 3.9, respectively).

Regarding the activity of the compounds against amastigote forms of *Lk* and *Lg*—two causative agents of cutaneous leishmaniasis predominantly in the Tunisian desert [42] and in French Guiana [43], respectively—caffeic acid (**34**) was the most active compound yielding the higher selective activity against *Lk* and *Lg* with IC_50_ values of 0.0039 and 0.0066 μM, respectively.

These results suggested the potential of **34** and its derivatives as promising compounds to be used as antileishmanials based on their high biological activity and the absence of toxicity against the host cells.

### 3.3. Antiplasmodial Activity

Malaria is a disease caused by different species of the genus *Plasmodium*. This disease is endemic in 91 countries and almost half of the world’s population is at risk of it [44], which means a dramatic global health issue. The rate of new cases fell by 37% globally during the period 2000–2015; however, the WHO still estimates that 214 million of cases of malaria and 438,000 deaths were reported in 2015 [44]. Although it does not belong to WHO’s list of NTDs, it represents a neglected disease in many parts of the world, where populations live in Malaria-infested regions under poor socio-economic conditions with no access to health services and adequate medication.

Malaria is a curable and preventable disease and the use of insecticide-treated mosquito nets is an essential and effective means to reduce the transmission and new infections. However, because of the increasing resistance of the parasites against the current therapies and the resistance of the mosquito against the available insecticides, the need of development of new therapies remains urgent.

The majority of results of research on antiplasmodial activity of *Salvia* species were obtained in studies with *Plasmodium falciparum*, the most lethal parasite responsible for Malaria tropica. No results on activity against other species of *Plasmodium* were found in the literature (Table 7 and Table 8).

#### 3.3.1. Extracts and Essential Oils

A series of seventeen extracts obtained with a mixture chloroform and methanol 1:1 (*v*/*v*) and eleven essential oils from seventeen different indigenous South African species of *Salvia* have been tested against the chloroquine-resistant *Pf* FCR-3 strain (Table 7) [45]. The IC_50_ values of the essential oils and extracts ranged from 1.2 to 13.50 µg/mL and 3.91 to 25.38 µg/mL, respectively. Most of the essential oils displayed better bioactivity than the solvent-extracts of the respective species. In this respect, the essential oil from *S. runcinata* L.f. (**v2**) and the chloroform-methanol extract of *S. radula Benth* (**z1**) represent the samples with the best biological activity (Table 7) [45].

Futhermore, *S. repens* Burch. ex Benth. and its extracts obtained with solvents of different polarity (named as **f**–**f5** in Table 7) have been extensively studied by different research groups. Kamatou and coworkers reported on the IC_50_ of a methanol extract of this plant with IC_50_ = 78.9 µg/mL [6]. Moreover, IC_50_ values for the chloroform-methanol (1:1) extract and also the essential oil of this species against chloroquine-sensitive *Pf* strain D10 were reported as 8.25 and 1.65 µg/mL, respectively [45]. Clarkson and coworkers tested the dichloromethane-methanol (1:1) extract of the whole plant against the chloroquine-sensitive strain (D10) of *Pf* resulting in an IC_50_ of 10.8 µg/mL [46]. The same extract was tested by Mokoka and colleagues against the chloroquine- and pyrimethamine-resistant K1 strain of *Pf* resulting IC_50_ values of 7.65 µg/mL and SI = 5.4 [6]. The results of the extracts thus indicated some significant differences.

Extracts of different polarity from three *Salvia* species were tested by Kirmizibekmez and coworkers against the resistant K1 strain and compared with the standard drug chloroquine [16]. The chloroform extracts of the three selected plants displayed the best IC_50_ values ranging from 2.54 to 3.72 µg/mL, the chloroform extract of *S. sclarea* L. (**b3**) being the most active one (Table 7).

Quite recently, Hammoud and coworkers tested different solvent extract of *Salvia chudaei* Batt. & Trab. and its essential oil against chloroquine resistance and sensitive *Pf* strains [47]. The essential oil (**Aa3**) showed the highest activity against both chloroquine resistance and sensitive parasites with IC_50_ of 2.39 ± 0.24, 2.40 ± 0.77 µg/mL, respectively, followed by the n-hexane extract (**Aa**) (IC_50_ = 4.91 ± 2.91 µg/mL against *Pf* K1 strain) (Table 7). No data about toxicity in mammalian cells were reported.

More studies to identify the compounds most probably responsible for the biological activity of these extracts are still required.

#### 3.3.2. Diterpenoids

Several *Salvia* diterpenoids have been reported to possess antiplasmodial activity. Diterpenes **10**–**17**, which also were tested against *Tbr* (see Section Diterpenoids) showed antiplasmodial activity with IC_50_ values ranging from 0.9 to 17.8 µM against *Pf* K1 with SI ranging from 0.1 to 18.2 [23] (Table 8). The mostly unfavorable values of selectivity call into question the security of most of these compounds and their use as lead compounds for new drugs. Only two compounds, Δ^9^-ferruginol (**14**) and ferruginol (**15**), were shown to be active and selective against *Pf* K1 strain. Both displayed IC_50_ values of 0.9 µM and SI values of 18.2 and 15.6, respectively, and may thus be considered promising hits [23]. Compound **15** was also isolated from the roots of *S. deserta* Schang. and tested against D6 chloroquine sensitive and W2 chloroquine resistance strains of *Pf* yielding IC_50_ values of 5.64 and 6.35 μM, respectively [39]. In this study, the ratio of cytotoxicity of Compound **15** against VERO cells over antiplasmodial activity was unfavorable with SI values of 0.8 and 0.7, respectively. This results contrasts with the results published by Ebrahimi et al. [23] (Table 8).

Ślusarczyk and colleagues isolated nine compounds from the dichloromethane extract of *S. miltiorrhiza* Bunge roots and identified them as tanshinone and abietane type diterpenoids (Compounds **3**–**9** and **41**–**42** in Table 8) [4]. They were tested against the chloroquine resistant K1 strain of *Pf*. The total extract showed 34% of growth inhibition at 0.81 µg/mL, while the isolated compounds displayed IC_50_ values ranging from 0.5 to ≥30 µM. Moreover, the SI values of these compounds were unfavorable, ranging from only 0.3 to 2.6. The relatively high activity of some of these compounds has been correlated with the presence of a furan D ring in their structure [4]. More investigations to relate structural elements with the activity and toxicity are necessary.

Another diterpene, betulafolientriol oxide (**43**), isolated from an active extract of *Salvia radula* Benth. (IC_50_ = 3.91 µg/mL, see **z1** in Table 7), showd antiplasmodial activity comparable with that of the crude extract, displaying an IC_50_ value of 10.39 μM against the FCR3 strain of *Pf* and a SI > 20, tested on kidney human cells [48].

In the same study cited above with respect to *Tbr* and *Tc* [24], Kuźma and coworkers tested in vitro the antiplasmodial activity of the four quinone methide-type diterpenes (**18**–**21** in Table 8) isolated from the roots of *S. austriaca* Jacq. against erythrocytic stages of *Pf* NF54. The IC_50_ values of the tested compound ranged from 1.9 µM to up to 167 µM, with taxodione (**18**) once more representing the most active compound. However, the SI of **18** was not favorable (SI = 1). In contrast, the antiplasmodial activity of Compound **18**—isolated from the roots of *S. deserta* Schang—was also determined against chloroquine sensitive (D6) and chloroquine resistant (W2) strains of *Pf* showing higher IC_50_ values (10.49 μM and 9.66 μM 3, respectively) [39].

Kuźma et al. also reported interesting biological activities of the diterpenes 7-(2′-oxohexyl)-taxodione (**20**) and taxadone (**21**), which demonstrated antiplasmodial activity at IC_50_ values of 3.37 and 3.66 µM, respectively. However, less promising was the antiplasmodial activity of 7-O-acetylhorminone (**30**) and horminone (**31**), for which the IC_50_ values were shown to be higher than 12.72 μM [39].

In view of the presented data it can be concluded that diterpenoids showed some favorable activity results but also present some toxicity to mammalian cells, which renders their direct usefulness in unmodified form as antimalarials unlikely. However, it might be useful to conduct further investigations, such as quantitative structure-activity relationship (QSAR) analysis with series of chemically modified compounds of this type, which could help to determine which characteristics and substituents of the compounds are most important to increase the antiplasmodial activity and their selectivity. A progress in this direction might make it possible to improve the properties of such natural products with respect to a more favorable activity/selectivity ratio.

#### 3.3.3. Triterpenes and Related Terpenoids

The two triterpenoids isolated from *S. hydrangea* DC. ex Benth., salvadione C (**22**) and perovskone B (**23**), were not only reported to show antitrypanosomal effects (see Section Triterpenoids) but also shown to be antiplasmodial agents (Table 8). The n-hexane extract of the plant (**d**) displayed an IC_50_ value of 3.2 µg/mL (Table 7) against *Pf* and the two isolated triterpenoids, **22** and **23**, were reported to have antiplasmodial activity with IC_50_ values of 1.43 and 0.18 µM, and SI values of 86.2 and 69.6, respectively [25]. Both the high bioactivity and the favorable SI values indicate that they can be considered as promising lead compounds to be further investigated. More research in terms of in vivo-activity and bioavailability as well as their mechanism of action should be performed.

The common triterpene oleanolic acid (**33**) (Table 8)—also isolated from the extract of *S. hydrangea* DC. ex Benth. (IC_50_ < 12.5 µg/mL)—displayed much lower activity with an IC_50_ value of 19.3 µM when tested against the 3D7 strain of *Pf* [8].

Farimani and colleagues also isolated from *S. hydrangea* DC. ex Benth. the unusual isoprenoid hydrangenone (**44**), which showed antiplasmodial activity with IC_50_ = 1.4 µM and a favorable SI = 6 [49].

#### 3.3.4. Flavonoids

Although the antiprotozoal activity of some flavonoids has been reported by different authors [40,50], little has been published in the case of *Salvia*. To the best of the knowledge, only the flavonoid salvigenin (**45**), extracted from the active extract from *S. radula* Benth. (IC_50_ = 3.91 µg/mL), was tested against *Pf*, yielding a low antiplasmodial activity (IC_50_ value 74.98 μM and SI > 4) [48].

## 4. Conclusions

The present review has summarized the current literature knowledge on the antiprotozoal activity of crude extracts from various *Salvia* species and of a variety of isolated compounds.

Relatively little has been reported on the traditional use of *Salvia* against the parasites under study. Thirty-five species of *Salvia*—or compounds islated from them—have been tested against at least one of these pathogens. Antiplasmodial activity was most frequently reported and antitrypanosomal activity against *T. cruzi* was the least well studied. However, the latter was the only parasite for which in vivo studies have been performed with a compound from *Salvia*.

From the 35 species, 45 compounds were isolated and have been tested against the protozoans treated in this review. Diterpenes of the abietane group represent the most active compounds against *T. brucei* and *Pf*, whereas phenolic compounds seem to show higher activity against *Leishmania* spp. The only flavonoid isolated so far from *Salvia* species—salvigenin (**45**)—and tested against protozoa did not show any promising results.

When inspecting the data summarized here, the bioactivity of the various extracts and compounds against the different parasites under study vary over a rather wide range and it is not trivial to make a judgement which level of activity should be considered strong or which entity could be promising for further studies. Naturally, the view on this issue of authors publishing such activity of their compounds will vary almost as widely as the data reported. Generally, from the authors’ own experience, *T. brucei* appears to be the most sensitive of the parasites treated here and *T. cruzi*, in particular if the clinically most relevant intracellular amastigote stage is assayed, is the hardest to kill in vitro. For a rough orientation, the IC_50_ values of standard positive controls routinely used in many studies, such as those conducted by our group in collaboration with the Swiss Tropical and Public Health Institute (Basel, Switzerland), are typically in the following ranges of magnitude: melarsoprol (*Tbr*, STIB 9000 strain, bloodstream trypomastigotes): 0.01 µM; benznidazole (*Tc*, Tulahuen C4 strain, intracellular amastigotes in L6 rat skeletal myoblasts): 4.5 µM; miltefosine (*Ld* MHOM-ET-67/L82, axenic amastigotes): 0.2 µM; and chloroquine (*Pf*, NF54 strain, intraerythrocytic forms): 0.02 µM (see e.g., [21]; data reported there are in µg/mL). It would appear overly critical, however, to concentrate only on such entities that outmatch these positive controls’ activity because all of them are clinically used drugs and the native natural products may only represent starting points for further optimization. Thus, in our own work, we would consider any isolated natural product that displays an IC_50_ value <0.1, <10, <2 and <1 µM against the mentioned forms of *Tbr*, *Tc*, *Ld* and *Pf*, respectively, a valuable hit for further studies, in case it also displays a favorable selectivity index (determined with L6 cells), e.g., >5–10.

Some of the reported compounds showed interesting in vitro biological activity, which could be considered a starting point for further investigations, such as structure-activity, mechanistic and in vivo studies. There appears to be a lack of further research-steps beyond the stage of in vitro phenotypic assays to exploit the full potential of these compounds in terms of the development of lead compounds into drugs. This may be due to the lack of funding within the current R&D system which is mainly guided by economical interest and often reluctant to support research in this field, which makes it difficult for many academic research groups to advance promising hits to further development steps. Most information on native natural compounds with promising activity that might be worth optimization is published at the stage of in vitro tests and then tends to be dissipated in the literature. The compounds often “vanish” in refrigerators in many laboratories around the globe but this valuable knowledge and material is not further followed up in a systematic way [7,40]. Further research steps such as a thorough investigation of structure-activity and structure-selectivity relationships, studies on the (possibly new) mechanisms of action and studies on the in vivo fate and efficacy of promising compounds often never happen. The information on *Salvia* metabolites and their antiprotozoal activity collected in this review will be useful for further studies on related species and their chemical constituents in the quest for new effective and affordable drugs against NTDs. A review such as this can help to avoid repetition of research, e.g., if it is used as a guide to dereplicate the pattern of constituents in further, hitherto unstudied *Salvia* species. Most importantly, however, it would be useful to stimulate further research on structure-activity relationship (SAR) studies for compounds of the classes treated here.

## Figures and Tables

**Table 1:** Biological activity of extracts from different species of *Salvia* against *Trypanosoma brucei* (n.d./n.f. = not determined/not found).

Parasite	Strain	Plant Species	IC_50_ Extract	Type of Solvent Extract	SI	Ref.
*Tbr*	STIB 900	*S. tomentosa* Mill. (**a1**)	3.64 µg/mL	MeOH-extract	24.73	[16]
*Tbr*	STIB 900	*S. tomentosa* Mill. (**a2**)	1.24 µg/mL	n-Hexane-extract	21.16	[16]
*Tbr*	STIB 900	*S. tomentosa* Mill. (**a3**)	2.33 µg/mL	Chloroform-extract	13.65	[16]
*Tbr*	STIB 900	*S. tomentosa* Mill. (**a4**)	10.96 µg/mL	H_2_O-extract	>8	[16]
*Tbr*	STIB 900	*S. sclarea* L. (**b1**)	6.44 µg/mL	MeOH-extract	13.6	[16]
*Tbr*	STIB 900	*S. sclarea* L. (**b2**)	2.4 µg/mL	n-Hexane-extract	7.63	[16]
*Tbr*	STIB 900	*S. sclarea* L. (**b3**)	4.4 µg/mL	Chloroform-extract	19.07	[16]
*Tbr*	STIB 900	*S. sclarea* L. (**b4**)	10.31 µg/mL	H_2_O-extract	>8.7	[16]
*Tbr*	STIB 900	*S. dichroantha* Stapf. (**c1**)	3.58 µg/mL	MeOH-extract	>25	[16]
*Tbr*	STIB 900	*S. dichroantha* Stapf. (**c2**)	3.5 µg/mL	n-Hexane-extract	>25.7	[16]
*Tbr*	STIB 900	*S. dichroantha* Stapf. (**c3**)	4.4 µg/mL	Chloroform-extract	19.27	[16]
*Tbr*	STIB 900	*S. dichroantha* Stapf. (**c4**)	7.77 µg/mL	H_2_O-extract	>11.5	[16]
*Tbr*	STIB 900	*S. hydrangea* DC. ex Benth. (**d**)	18 µg/mL	n-Hexane-extract	n.d.	[25]
*Tbr*	STIB 900	*S. miltiorrhiza* Bunge (**e**)	97% inhibition at 0.81 µg/mL	DCM-extract	n.d.	[4]
*Tbr*	STIB 900	*S. repens* Burch.ex Benth. (**f**)	10.8 µg/mL	DCM-MeOH (1:1)	3.82	[26]
*Tbb*	Strain 427	*S. spathacea* Greene (**g**)	1.13 ± 0.78 µg/mL	Ethanolic extract	n.d.	[19]
*Tbb*	n.d.	*S. lavandulifolia* Vahl. (**h**)	No activity at >20 mg/mL	Essential oil	n.d.	[20]
*Tbr*	STIB 900	*S. officinalis* L. (**i**)	1.86 µg/mL	Ethanolic tincture	17.3	[21]

**Table 2:** Biological activity of extracts of *Salvia* spp. and their isolated compounds against *Trypanosoma brucei*, (n.d./n.f.d. = not determined/not found).

Parasite	Form/Strain	Plant Species	IC_50_ Extract	Isolated Compound	Molecular Formula	Molar Mass (g/mol)	IC_50_ of the Compound (µM)	SI	Ref
*Tbb*	GUT at 3.1	*S. leucantha* Cav.	n.d./n.f.	salviandulin E (**1**)	C_20_H_16_O_6_	352.09	2.04 µM (0.72 µg/mL)	1.17	[22]
*Tbb*	GUT at 3.1	*S. leucantha* Cav.	n.d./n.f.	6,7-dehydrodugesin A (**2**)	C_20_H_16_O_5_	336.09	>37.19 µM (>12.5 µg/mL)	n.d.	[22]
*Tbr*	Trypomastigotes/ STIB 900	*S. miltiorrhiza* Bunge.	97% inhib. at 0.81 µg/mL	miltirone (**3**)	C_19_H_22_O_2_	282.16	0.5 µM	2.6	[4]
*Tbr*	Trypomastigotes/ STIB 900	*S. miltiorrhiza* Bunge.	97% inhib. at 0.81 µg/mL	methylenetanshinquinone (**4**)	C_18_H_14_O_3_	278.09	0.5 µM	24.2	[4]
*Tbr*	Trypomastigotes/ STIB 900	*S. miltiorrhiza* Bunge.	97% inhib. at 0.81 µg/mL	tanshinone I (**5**)	C_18_H_12_O_3_	276.08	1.3 µM	9.5	[4]
*Tbr*	Trypomastigotes/ STIB 900	*S. miltiorrhiza* Bunge.	97% inhib. at 0.81 µg/mL	tanshinone IIa (**6**)	C_19_H_18_O_3_	294.13	1.5 µM	5	[4]
*Tbr*	Trypomastigotes/ STIB 900	*S. miltiorrhiza* Bunge.	97% inhib. at 0.81 µg/mL	methyl-tanshinonate (**7**)	C_20_H_18_O_5_	338.12	17 µM	0.4	[4]
*Tbr*	Trypomastigotes/ STIB 900	*S. miltiorrhiza* Bunge.	97% inhib. at 0.81 µg/mL	11-hydroxymiltiodiol (**8**)	C_19_H_22_O_3_	298.16	3.6 µM	n.d.	[4]
*Tbr*	Trypomastigotes/ STIB 900	*S. miltiorrhiza* Bunge.	97% inhib. at 0.81 µg/mL	cryptotanshinone (**9**)	C_19_H_20_O_3_	296.14	26.2 µM	0.2	[4]
*Tbr*	Trypomastigotes/ STIB 900	*S. sahendica* Boiss. & Buhse	n.d./n.f.	12-deoxy-salvipisone (**10**)	C_20_H_24_O_2_	296.18	2.5 µM	0.2	[23]
*Tbr*	Trypomastigotes/ STIB 900	*S. sahendica* Boiss. & Buhse	n.d./n.f.	sahandinone (**11**)	C_20_H_24_O_2_	296.18	1.8 µM	0.2	[23]
*Tbr*	Trypomastigotes/ STIB 900	*S. sahendica* Boiss. & Buhse	n.d./n.f.	12-deoxy-6,7-dehydroroyleanone (**12**)	C_20_H_26_O_2_	298.19	32.3 µM	0.9	[23]
*Tbr*	Trypomastigotes/ STIB 900	*S. sahendica* Boiss. & Buhse	n.d./n.f.	7α-acetoxyroyleanone (**13**)	C_22_H_30_O_5_	374.21	2.9 µM	0.1	[23]
*Tbr*	Trypomastigotes/ STIB 900	*S. sahendica* Boiss. & Buhse	n.d./n.f.	Δ 9-ferruginol (**14**)	C_20_H_28_O	284.21	12.8 µM	1.2	[23]
*Tbr*	Trypomastigotes/ STIB 900	*S. sahendica* Boiss. & Buhse	n.d./n.f.	ferruginol (**15**)	C_20_H_30_O	286.23	28.1 µM	0.5	[23]
*Tbr*	Trypomastigotes/ STIB 900	*S. sahendica* Boiss. & Buhse	n.d./n.f.	sahandol (**16**)	C_20_H_24_O_2_	296.18	18.4 µM	0.8	[23]
*Tbr*	Trypomastigotes/ STIB 900	*S. sahendica* Boiss. & Buhse	n.d./n.f.	sahandone (**17**)	C_21_H_26_O_3_	326.19	19.5 µM	0.6	[23]
*Tbr*	STIB 900	*S.austriaca* Jacq.	n.d./n.f.	taxodione (**18**)	C_20_H_26_O_3_	314.19	0.05 µM	38	[24]
*Tbr*	STIB 900	*S.austriaca* Jacq.	n.d./n.f.	15-deoxy-fuerstione (**19**)	C_20_H_26_O_2_	298.19	194.7 µM	0.69	[24]
*Tbr*	STIB 900	*S.austriaca* Jacq.	n.d./n.f.	7-(2′-oxohexyl)-taxodione (**20**)	C_26_H_36_O_4_	412.26	0.62 µM	5.0	[24]
*Tbr*	STIB 900	*S.austriaca* Jacq.	n.d./n.f.	taxodone (**21**)	C_20_H_28_O_3_	316.20	1.67 µM	2.4	[24]
*Tbr*	STIB 900	*S. hydrangea* DC. Ex Benth.	18 µg/mL	salvadione C (**22**)	C_30_H_40_O_5_	480.29	4.33 µM	43.2	[25]
*Tbr*	STIB 900	*S. hydrangea* DC. Ex Benth.	18 µg/mL	perovskone B (**23**)	C_30_H_40_O_4_	464.29	15.92 µM	0.78	[25]
*Tbr*	STIB 900	*S. urmiensis* Bunge.	n.d./n.f.	urmiensolide (**24**)	C_30_H_46_O_5_	486.33	5.6 μM	33	[17]

**Table 3:** Biological activity of extracts from different species of *Salvia* against *Trypanosoma cruzi*.

Parasite	Form/Strain	Plant Species	IC_50_ Extract	Type of Solvent Extract	SI	Ref.
*Tc*	Trypomastigote/Tulahuen strain C2C4	*S. tomentosa* Mill. (**a1**)	>90 µg/mL	MeOH-extract	<1	[16]
*Tc*	Trypomastigote/Tulahuen strain C2C4	*S. tomentosa* Mill. (**a2**)	28.46 µg/mL	n-Hexane-extract	0.92	[16]
*Tc*	Trypomastigote/Tulahuen strain C2C4	*S. tomentosa* Mill. (**a3**)	35.72 µg/mL	Chloroform-extract	0.89	[16]
*Tc*	Trypomastigote/Tulahuen strain C2C4	*S. tomentosa* Mill. (**a4**)	>90 µg/mL	H_2_O-extract	≈1	[16]
*Tc*	Trypomastigote/Tulahuen strain C2C4	*S. sclarea* L. (**b1**)	56.82 µg/mL	MeOH-extract	1.54	[16]
*Tc*	Trypomastigote/Tulahuen strain C2C4	*S. sclarea* L. (**b2**)	18.17 µg/mL	n-Hexane-extract	1	[16]
*Tc*	Trypomastigote/Tulahuen strain C2C4	*S. sclarea* L. (**b3**)	52.51 µg/mL	Chloroform-extract	1.59	[16]
*Tc*	Trypomastigote/Tulahuen strain C2C4	*S. sclarea* L. (**b4**)	>90 µg/mL	H_2_O-extract	≈1	[16]
*Tc*	Trypomastigote/Tulahuen strain C2C4	*S. dichroantha* Stapf. (**c1**)	>90 µg/mL	MeOH-extract	≈1	[16]
*Tc*	Trypomastigote/Tulahuen strain C2C4	*S. dichroantha* Stapf. (**c2**)	41.85 µg/mL	n-Hexane-extract	>2.15	[16]
*Tc*	Trypomastigote/Tulahuen strain C2C4	*S. dichroantha* Stapf. (**c3**)	48.99 µg/mL	Chloroform-extract	1.73	[16]
*Tc*	Trypomastigote/Tulahuen strain C2C4	*S. dichroantha* Stapf. (**c4**)	>90 µg/mL	H_2_O-extract	≈1	[16]
*Tc*	Trypomastigote/Tulahuen strain C2C4	*S. repens* Burch.ex Benth. (**f**)	36.2 µg/mL	DCM-MeOH (1:1)	1.15	[26]

**Table 4:** Biological activity of extracts of *Salvia* spp. and their isolated compounds against *Trypanosoma cruzi*. (n.d.= not determined).

Parasite	Form/Strain	Plant Species	IC_50_ Extract	Isolated Compound	Molecular Formula	Molar Mass (g/mol)	IC_50_ of the Compound (µM)	SI	Ref.
*Tc*	Epimastigotes/Tulhauen	*S. gilliessi* Benth.	n.d.	5-epi-icetexone (ICTX) (**25**)	C_20_H_22_O_5_	342.15	6.5 ± 0.75 µM	n.d.	[27]
*Tc*	Amastigotes/Y Strain	*S. gilliessi* Benth.	n.d.	5-epi-icetexone (ICTX) (**25**)	C_20_H_22_O_5_	342.15	n.d.	n.d.	[28]
*Tc*	Epimastigotes/Dm28c strain	*S. cuspidata* (Ruiz & Pav. Subsp. gilliesii (Benth.) J.R.I. Wood	n.d.	12-hydroxy-11,14-diketo-6,8,12-abietatrien-19,20-olide (HABTO) (**26**)	C_17_H_16_O_5_	300.10	≈16.6 µM (≈5 µg/mL)	n.d.	[30]
*Tc*	Amastigotes/Tulahuen strain C2C4 containing Lac Z	*S. austriaca* Jacq.	n.d.	taxodione (**18**)	C_20_H_26_O_3_	314.19	7.11 µM	0.27	[24]
*Tc*	Amastigotes/Tulahuen strain C2C4 containing Lac Z	*S. austriaca* Jacq.	n.d.	15-Deoxy-fuerstione (**19**)	C_20_H_26_O_2_	298.19	146.9 µM	0.91	[24]
*Tc*	Amastigotes/Tulahuen strain C2C4 containing Lac Z	*S. austriaca* Jacq.	n.d.	7-(2′-oxohexyl)-taxodione (**20**)	C_26_H_36_O_4_	412.26	7.76 µM	0.4	[24]
*Tc*	Amastigotes/Tulahuen strain C2C4 containing Lac Z	*S. austriaca* Jacq.	n.d.	taxodone (**21**)	C_20_H_28_O_3_	316.20	7.63 µM	0.5	[24]

**Table 5:** Biological activity of extracts from different species of *Salvia* against *Leishmania* spp. (n.d./n.f. = not determined/not found). *.

Parasite	Form/Strain	Plant Species	IC_50_ Extract	Type of Solvent Extract	SI	Ref.
*n.d.*	(Popular use antileishmanial remedy)	*S. hydrangea* DC ex. Benth. (**d1**)	n.d.	n.d.	n.d.	[8]
*Ld*	Axenic amastigotes/ strain MHOM/ET/67/L82	*S. tomentosa* Mill. (**a1**)	14.92 µg/mL	MeOH-extract	24.73	[16]
*Ld*	Axenic amastigotes/ strain MHOM/ET/67/L82	*S. tomentosa* Mill. (**a2**)	2.49 µg/mL	n-Hexane-extract	21.16	[16]
*Ld*	Axenic amastigotes/ strain MHOM/ET/67/L82	*S. tomentosa* Mill. (**a3**)	1.81 µg/mL	Chloroform-extract	29.47	[16]
*Ld*	Axenic amastigotes/ strain MHOM/ET/67/L82	*S. tomentosa* Mill. (**a4**)	>90 µg/mL	H_2_O-extract	>10	[16]
*Ld*	Axenic amastigotes/ strain MHOM/ET/67/L82	*S. sclarea* L. (**b1**)	12.95 µg/mL	MeOH-extract	13.6	[16]
*Ld*	Axenic amastigotes/ strain MHOM/ET/67/L82	*S. sclarea* L. (**b2**)	5.25 µg/mL	n-Hexane-extract	7.63	[16]
*Ld*	Axenic amastigotes/ strain MHOM/ET/67/L82	*S. sclarea* L. (**b3**)	8.31 µg/mL	Chloroform-extract	19.07	[16]
*Ld*	Axenic amastigotes/ strain MHOM/ET/67/L82	*S. sclarea* L. (**b4**)	47.88 µg/mL	H_2_O-extract	>9	[16]
*Ld*	Axenic amastigotes/ strain MHOM/ET/67/L82	*S. dichroantha* Stapf. (**c1**)	4.93 µg/mL	MeOH-extract	>25	[16]
*Ld*	Axenic amastigotes/ strain MHOM/ET/67/L82	*S. dichroantha* Stapf. (**c2**)	3.48 µg/mL	n-Hexane-extract	25.7	[16]
*Ld*	Axenic amastigotes/ strain MHOM/ET/67/L82	*S. dichroantha* Stapf. (**c3**)	2.31 µg/mL	Chloroform-extract	19.27	[16]
*Ld*	Axenic amastigotes/ strain MHOM/ET/67/L82	*S. dichroantha* Stapf. (**c4**)	>90 µg/mL	H_2_O-extract	>11	[16]
*Ld*	Axenic amastigotes/ strain MHOM/ET/67/L82	*S. repens* Burch. ex Benth. (**f**)	5.36 µg/mL	DCM-MeOH (1:1)	7.74	[26]
*Ld*	Promastigotes	*S. lavandulifolia* Vahl. (**h**)	No activity up to 20 µg/mL	Essential oil	n.d.	[20]
*Ld*	Axenic amastigotes	*S. lavandulifolia* Vahl. (**h**)	No activity up to 20 µg/mL	Essential oil	n.d.	[20]
*Ld*	Intracellular amastigotes	*S. lavandulifolia* Vahl. (**h**)	No activity up to 20 µg/mL	Essential oil	n.d.	[20]
*Lm*	Promastigote/ LCO3	*S.officinalis* L. (**i**)	3.40 ± 0.16 µg/mL	Essential oil	5.92	[34]
*Li*	Promastigote/ LV20	*S.officinalis* L. (**i1**)	2.67 ± 0.33 µg/mL	Essential oil	7.54	[34]
*Lm*	Promastigotes/ MROH/IR/75/IR	*S.officinalis* L. (**i2**)	184 ± 11.17 µg/mL	MeOH-maceration	n.d.	[35]
*Lm*	Amastogotes	*S.officinalis* L. (**i2**)	58% letally at 184 ± 11.17 µg/mL	MeOH-maceration	n.d.	[35]
*Lm*	Promastigotes	*S. bucharica* Popov (**j**)	72.31 µg/mL	MeOH-extract	n.d.	[36]
*Lm*	Promastigotes	*S. bucharica* Popov (**j1**)	50.51 µg/mL	Chloroform-extract	n.d.	[36]
*Lm*	Promastigotes	*S. bucharica* Popov (**j2**)	>100 µg/mL	Acetone-extract	n.d.	[36]
*Lm*	Promastigotes	*S. bucharica* Popov (**j3**)	30.51 µg/mL	H_2_O extract	n.d.	[36]
*Lm*	Promastigotes/ MHOM/MA/2009/LCER19-09	*S. verbenaca* (L.) Briq. ssp *verbenaca* Maire (**k**)	155.43 µg/mL	n-Hexane-extract	n.d.	[37]
*Lm*	Promastigotes/ MHOM/MA/2009/LCER19-09	*S. verbenaca* (L.) Briq. ssp *verbenaca* Maire (**k1**)	24.56 µg/mL	DCM-extract	n.d.	[37]
*Lm*	Promastigotes/ MHOM/MA/2009/LCER19-09	*S. verbenaca* (L.) Briq. ssp *verbenaca* Maire (**k2**)	>1000 µg/mL	MeOH-extract	n.d.	[37]
*Li*	Promastigotes/ MHOM/MA/1998/LVTA	*S. verbenaca* (L.) Briq. ssp *verbenaca* Maire (**k**)	14.11 µg/mL	n-Hexane-extract	n.d.	[37]
*Li*	Promastigotes/ MHOM/MA/1998/LVTA	*S. verbenaca* (L.) Briq. ssp *verbenaca* Maire (**k1**)	31.57 µg/mL	DCM-extract	n.d.	[37]
*Li*	Promastigotes/ MHOM/MA/1998/LVTA	*S. verbenaca* (L.) Briq. ssp *verbenaca* Maire (**k2**)	>1000 µg/mL	MeOH-extract	n.d.	[37]
*Lt*	Promastigotes/ MHOM/MA/2010/LCTIOK-4	*S. verbenaca* (L.) Briq. ssp *verbenaca* Maire (**k**)	148.23 µg/mL	n-Hexane-extracts	n.d.	[37]
*Lt*	Promastigotes/ MHOM/MA/2010/LCTIOK-4	*S. verbenaca* (L.) Briq. ssp *verbenaca* Maire (**k1**)	33.77 µg/mL	DCM-extract	n.d.	[37]
*Lt*	Promastigotes/ MHOM/MA/2010/LCTIOK-4	*S. verbenaca* (L.) Briq. ssp *verbenaca* Maire (**k2**)	>1000 µg/mL	MeOH-extract	n.d.	[37]

* Leishmania donovani (*Ld*), *Leishmania major* (*Lm*), *Leishmania infantum* (*Li*), *Leishmania guyanensis* (*Lg*), *Leishnmania killicki* (*Lk*), and *Leishmania tropica* (*Lt*).

**Table 6:** Biological activity of extracts of *Salvia* spp. and their isolated compounds against *Leishmania* spp. (n.d./n.f. = not determined/not found). *

Parasite	Form/Strain	Plant Species	IC_50_ Extract	Isolated Compound	Molecular Formula	Molar Mass (g/mol)	IC_50_ of the Compound	SI	Ref.
*Ld*	Axenic amastigotes/ MHOM/ET/67/L82	*S.repens* Burch.ex Benth.	5.36 µg/mL	12-methoxycarnosic acid (**27**)	C_21_H_30_O_4_	346.21	0.75 μM	23.06	[18]
*Ld*	Intracellular Amastgitotes /n.d.	*S. cilicica* Boiss and Kotschy	n.d.	7-hydroxy-12-methoxy-20-nor-abieta-1,5(10),7,9,12-pentaen-6,14-dione (**28**)	C_20_H_22_O_4_	326.15	0.17 μM (170 nM)	>1.76	[38]
*Ld*	Promastigotes/n.d.	*S. cilicica* Boiss and Kotschy	n.d.	7-hydroxy-12-methoxy-20-nor-abieta-1,5(10),7,9,12-pentaen-6,14-dione (**28**)	C_20_ H_22_ O_4_	326.15	>0.3 μM (>300 nM)	≈1	[38]
*Lm*	Amastigotes/n.d.	*S. cilicica* Boiss and Kotschy	n.d.	7-hydroxy-12-methoxy-20-nor-abieta-1,5(10),7,9,12-pentaen-6,14-dione (**28**)	C_20_H_22_O_4_	326.15	0.287 μM (287.4 nM)	1.04	[38]
*Lm*	Promastigotes/n.d.	*S. cilicica* Boiss and Kotschy	n.d.	7-hydroxy-12-methoxy-20-nor-abieta-1,5(10),7,9,12-pentaen-6,14-dione (**28**)	C_20_H_22_O_4_	326.15	≥0.3 μM (≥300 nM)	≈1	[38]
*Ld*	Amastgitotes/n.d.	*S. cilicica* Boiss and Kotschy	n.d.	abieta-8,12-dien-11,14-dione (12-deoxy-royleanone) (**29**)	C_20_H_28_O_2_	300.21	0.121 μM (121 nM)	1.58	[38]
*Ld*	Promastigotes/n.d.	*S. cilicica* Boiss and Kotschy	n.d.	abieta-8,12-dien-11,14-dione (12-deoxy-royleanone) (**29**)	C_20_H_28_O_2_	300.21	>0.3 μM (>300 nM)	≤0.64	[38]
*Lm*	Amastigotes/n.d.	*S. cilicica* Boiss and Kotschy	n.d.	abieta-8,12-dien-11,14-dione (12-deoxy-royleanone) (**29**)	C_20_H_28_O_2_	300.21	0.182 μM (182.3 nM)	1.04	[38]
*Lm*	Promastigotes/n.d.	*S. cilicica* Boiss and Kotschy	n.d.	abieta-8,12-dien-11,14-dione (12-deoxy-royleanone) (**29**)	C_20_H_28_O_2_	300.21	≥0.30 μM (≥300 nM)	≤0.63	[38]
*Ld*	Promastigotes/n.d.	*S. deserta* Schang.	n.d.	taxodione (**18**)	C_20_H_26_O_3_	314.42	1.46 ± 0.52 μM	10.34	[39]
*Ld*	Promastigotes/n.d.	*S. deserta* Schang.	n.d.	ferruginol (**15**)	C_20_H_30_O	286.45	11.39 ± 1.05 μM	n.d.	[39]
*Ld*	Promastigotes/n.d.	*S. deserta* Schang.	n.d.	7-O-acetylhorminone (**30**)	C_22_H_30_O_5_	374.47	19.69 ± 0.80 μM	n.d.	[39]
*Ld*	Promastigotes/n.d.	*S. deserta* Schang.	n.d.	horminone (**31**)	C_20_H_28_O_4_	332.20	29.43 ± 3.01 μM	n.d.	[39]
*Ld*	Amastgitotes/n.d.	*S. cilicica* Boiss and Kotschy	n.d.	ursolic acid (**32**)	C_30_H_48_O_3_	456.36	0.0127 μM (12.7 nM)	2.22	[38]
*Ld*	Promastigotes/n.d.	*S. cilicica* Boiss and Kotschy	n.d.	ursolic acid (**32**)	C_30_H_48_O_3_	456.36	0.091 μM (91 nM)	0.17	[38]
*Lm*	Promastigotes/n.d.	*S. cilicica* Boiss and Kotschy	n.d.	ursolic acid (**32**)	C_30_H_48_O_3_	456.36	0.051 μM (51.3 nM)	0.3	[38]
*Lm*	Amastigotes/n.d.	*S. cilicica* Boiss and Kotschy	n.d.	ursolic acid (**32**)	C_30_H_48_O_3_	456.36	0.007 μM (7 nM)	2.22	[38]
*Ld*	Promastigotes/n.d.	*S. cilicica* Boiss and Kotschy	n.d.	oleanolic acid (**33**)	C_30_H_48_O_3_	456.36	0.091 μM (91 nM)	1.45	[38]
*Ld*	Amastigotes/n.d.	*S. cilicica* Boiss and Kotschy	n.d.	oleanolic acid (**33**)	C_30_H_48_O_3_	456.36	0.063 μM (62.9 nM)	2.1	[38]
*Lm*	Promastigotes/n.d.	*S. cilicica* Boiss and Kotschy	n.d.	oleanolic acid (**33**)	C_30_H_48_O_3_	456.36	0.137 μM (137 nM)	0.97	[38]
*Lm*	Amastigotes	*S. cilicica* Boiss and Kotschy	n.d.	oleanolic acid (**33**)	C_30_H_48_O_3_	456.36	0.120 μM (119.9 nM)	1.1	[38]
*Lm*	Amastigotes/ LV39 strain	*S. officinalis* L.	n.d.	caffeic acid (**34**)	C_9_H_8_O_4_	180.04	0.0044 μM (4.4 nM)	>2200	[41]
*Ld*	Amastigotes/ LV9 strain	*S. officinalis* L.	n.d.	caffeic acid (**34**)	C_9_H_8_O_4_	180.04	0.0061 μM (6.1 nM)	>500	[41]
*Lg*	Amastigotes	*S. officinalis* L.	n.d.	caffeic acid (**34**)	C_9_H_8_O_4_	180.04	0.0066 μM (6.6 nM)	>360	[41]
*Lk*	Amastigotes	*S. officinalis* L.	n.d.	caffeic acid (**34**)	C_9_H_8_O_4_	180.04	0.0039 μM (3.9 nM)	>333	[41]
*Lm/Ld/* *Lk/Lg*	Promastigotes	*S. officinalis* L.	n.d.	caffeic acid (**34**)	C_9_H_8_O_4_	180.04	>2.8 μM (>2800 nM)	≈0.78	[41]
*Lm*	Amastigotes/ LV39 strain	*S. officinalis* L.	n.d.	rosmarinic acid (**35**)	C_18_H_16_O_8_	360.08	0.0592 μM (59.2 nM)	>18	[41]
*Ld*	Amastigotes/ LV9 strain	*S. officinalis* L.	n.d.	rosmarinic acid (**35**)	C_18_H_16_O_8_	360.08	0.0744 μM (74.4 nM)	>15	[41]
*Lg*	Amastigotes	*S. officinalis* L.	n.d.	rosmarinic acid (**35**)	C_18_H_16_O_8_	360.08	0.0842 μM (84.2 nM)	>13	[41]
*Lk*	Amastigotes	*S. officinalis* L.	n.d.	rosmarinic acid (**35**)	C_18_H_16_O_8_	360.08	0.0694 μM (69.4 nM)	>15	[41]
*Lm/Ld/* *Lk/Lg*	Promastigote	*S. officinalis* L.	n.d.	rosmarinic acid (**35**)	C_18_H_16_O_8_	360.08	>1.4 μM (>1400 nM)	≈0.78	[41]
*Lm*	Amastigotes/ LV39 strain	*S. officinalis* L.	n.d.	salvianolic acid I (**36**)	C_27_H_22_O_12_	538.11	0.1604 μM (160.4 nM)	> 4.35	[41]
*Ld*	Amastigotes/ LV9 strain	*S. officinalis* L.	n.d.	salvianolic acid I (**36**)	C_27_H_22_O_12_	538.11	0.1758 μM (175.8 nM)	>3.9	[41]
*Lg*	Amastigotes	*S. officinalis* L.	n.d.	salvianolic acid I (**36**)	C_27_H_22_O_12_	538.11	0.1515 μM (151.5 nM)	>4.62	[41]
*Lk*	Amastigotes	*S. officinalis* L.	n.d.	salvianolic acid I (**36**)	C_27_H_22_O_12_	538.11	0.1678 μM (167.8 nM)	>4.17	[41]
*Lm/Ld/* *Lk/Lg*	Promastigote	*S. officinalis* L.	n.d.	salvianolic acid I (**36**)	C_27_H_22_O_12_	538.11	>0.9 μM (>900 nM)	≈0.77	[41]
*Lm*	Amastigotes/strain	*S. officinalis* L.	n.d.	methyl ester of salvianolic acid I (**37**)	C_28_H_24_O_12_	552.13	0.0108 μM (10.8 nM)	>64.81	[41]
*Ld*	Amastigotes	*S. officinalis* L.	n.d.	methyl ester of salvianolic acid I (**37**)	C_28_H_24_O_12_	552.13	0.0186 μM (18.6 nM)	>37.63	[41]
*Lg*	Amastigotes	*S. officinalis* L.	n.d.	methyl ester of salvianolic acid I (**37**)	C_28_H_24_O_12_	552.13	0.0152 μM (15.2 nM)	>46	[41]
*Lk*	Amastigotes	*S. officinalis* L.	n.d.	methyl ester of salvianolic acid I (**37**)	C_28_H_24_O_12_	552.13	0.0136 μM (13.6 nM)	>51.47	[41]
*Lm/Ld/* *Lk/Lg*	Promastigote	*S. officinalis* L.	n.d.	methyl ester of salvianolic acid I (**37**)	C_28_H_24_O_12_	552.13	>0.9 μM (>900 nM)	≈0.77	[41]
*Lm*	Amastigotes/ LV39 strain	*S. officinalis* L.	n.d.	salvianolic acid K (**38**)	C_27_H_24_O_13_	556.12	0.0183 μM (18.3 nM)	>38.25	[41]
*Ld*	Amastigotes/ LV9 strain	*S. officinalis* L.	n.d.	salvianolic acid K (**38**)	C_27_H_24_O_13_	556.12	0.0182 μM (18.2 nM)	>38.46	[41]
*Lg*	Amastigotes	*S. officinalis* L.	n.d.	salvianolic acid K (**38**)	C_27_H_24_O_13_	556.12	0.0133 μM (13.3 nM)	>52.63	[41]
*Lk*	Amastigotes	*S. officinalis* L.	n.d.	salvianolic acid K (**38**)	C_27_H_24_O_13_	556.12	0.0145 μM (14.5 nM)	>48.27	[41]
*Lm/Ld/* *Lk/Lg*	Promastigote	*S. officinalis* L.	n.d.	salvianolic acid K (**38**)	C_27_H_24_O_13_	556.12	>0.9 μM (>900 nM)	≈0.77	[41]
*Lm*	Amastigotes/ LV39 strain	*S. officinalis* L.	n.d.	salvianolic acid L (**39**)	C_36_H_30_O_16_	718.15	0.0203 μM (20.3 nM)	>34,48	[41]
*Ld*	Amastigotes/ LV9 strain	*S. officinalis* L.	n.d.	salvianolic acid L (**39**)	C_36_H_30_O_16_	718.15	0.0154 μM (15.4 nM)	>45.45	[41]
*Lg*	Amastigotes	*S. officinalis* L.	n.d.	salvianolic acid L (**39**)	C_36_H_30_O_16_	718.15	0.0226 μM (22.6 nM)	>30.97	[41]
*Lk*	Amastigotes	*S. officinalis* L.	n.d.	salvianolic acid L (**39**)	C_36_H_30_O_16_	718.15	0.013 μM (13.0 nM)	>53.84	[41]
*Lm/Ld/* *Lk/Lg*	Promastigote	*S. officinalis* L.	n.d.	salvianolic acid L (**39**)	C_36_H_30_O_16_	718.15	>0.9 μM (>900 nM)	≈0.77	[41]
*Lm*	Amastigotes/ LV39 strain	*S. officinalis* L.	n.d.	sagerinic acid (**40**)	C_36_H_32_O_16_	720.17	0.128 μM (128.7 nM)	>4.66	[41]
*Ld*	Amastigotes/ LV9 strain	*S. officinalis* L.	n.d.	sagerinic acid (**40**)	C_36_H_32_O_16_	720.17	0.122 μM (122.1 nM)	>4.91	[41]
*Lg*	Amastigotes	*S. officinalis* L.	n.d.	sagerinic acid (**40**)	C_36_H_32_O_16_	720.17	0.142 μM (141.5 nM)	>4.24	[41]
*Lk*	Amastigotes	*S. officinalis* L.	n.d.	sagerinic acid (**40**)	C_36_H_32_O_16_	720.17	0.155 μM (154.8 nM)	>3.88	[41]
*Lm/Ld/* *Lk/Lg*	Promastigote	*S. officinalis* L.	n.d.	sagerinic acid (**40**)	C_36_H_32_O_16_	720.17	>0.7 μM (>700 nM)	≈0.85	[41]

* Leishmania donovani (*Ld*), *Leishmania major* (*Lm*), *Leishmania infantum* (*Li*), *Leishmania guyanensis* (*Lg*), *Leishnmania killicki* (*Lk*), and *Leishmania tropica* (*Lt*).

**Table 7:** Biological activity of extracts and essential oils of *Salvia* spp. against *P. falciparum* (n.d./n.f. = not determined/not found).

Parasite	Strain	Plant Species	Type of Solvent Extract	IC_50_ Solvent Extract	SI	Ref.
*Pf*	Chloroquine- and Pyrimethamine-resistant K1 strain	*S. tomentosa* Mill. (**a1**)	MeOH-extract	9.94 µg/mL	9.05	[16]
*Pf*	Chloroquine- and Pyrimethamine-resistant K1 strain	*S. tomentosa* Mill. (**a2**)	n-Hexane-extract	3.47 µg/mL	7.56	[16]
*Pf*	Chloroquine- and Pyrimethamine-resistant K1 strain	*S. tomentosa* Mill. (**a3**)	Chloroform-extract	3.14 µg/mL	10.12	[16]
*Pf*	Chloroquine- and Pyrimethamine-resistant K1 strain	*S. tomentosa* Mill. (**a4**)	H_2_O-extract	>20 µg/mL	≈4.5	[16]
*Pf*	Chloroquine- and Pyrimethamine-resistant K1 strain	*S. sclarea* L. (**b1**)	MeOH-extract	6.6 µg/mL	13.27	[16]
*Pf*	Chloroquine- and Pyrimethamine-resistant K1 strain	*S. sclarea* L. (**b2**)	n-Hexane-extract	3.78 µg/mL	4.84	[16]
*Pf*	Chloroquine- and Pyrimethamine-resistant K1 strain	*S. sclarea* L. (**b3**)	Chloroform-extract	2.54 µg/mL	33.03	[16]
*Pf*	Chloroquine- and Pyrimethamine-resistant K1 strain	*S. sclarea* L. (**b4**)	H_2_O-extract	>20 µg/mL	≈4.5	[16]
*Pf*	Chloroquine- and Pyrimethamine-resistant K1 strain	*S. dichroantha* Stapf. (**c1**)	MeOH-extract	8.85 µg/mL	>10.17	[16]
*Pf*	Chloroquine- and Pyrimethamine-resistant K1 strain	*S. dichroantha* Stapf. (**c2**)	n-Hexane-extract	4.17 µg/mL	>21.58	[16]
*Pf*	Chloroquine- and Pyrimethamine-resistant K1 strain	*S. dichroantha* Stapf. (**c3**)	Chloroform-extract	3.72 µg/mL	22.79	[16]
*Pf*	Chloroquine- and Pyrimethamine-resistant K1 strain	*S. dichroantha* Stapf. (**c4**)	H_2_O-extract	>20 µg/mL	≈4.5	[16]
*Pf*	K1 strain	*S. hydrangea* DC ex. Benth. (**d**)	n-Hexane-extract	3.2 µg/mL	n.d.	[25]
*Pf*	3D7 strain	*S. hydrangea* DC ex. Benth. (**d2**)	n.d.	<12.5 µg/mL	n.d.	[8]
*Pf*	K1 strain	*S. repens* Burch. ex Benth. (**f**)	DCM/MeOH 1:1	7.65 µg/mL	5.4	[26]
*Pf*	Chloroquine-resistant *Pf* FCR-3 strain	*S. repens* Burch. ex Benth. (**f1**)	Chloroform/MeOH (1:1)	8.25 µg/mL	n.d.	[45]
*Pf*	Chloroquine-resistant *Pf* FCR-3 strain	*S. repens* Burch. ex Benth. (**f2**)	Essential oil	1.65 µg/mL	n.d.	[45]
*Pf*	Chloroquine-sensitive strain D10	*S. repens* Burch. ex Benth. (**f3**)	DCM/MeOH 1:1	10.8 µg/mL	n.d.	[46]
*Pf*	n.d.	*S. repens* Burch. ex Benth. (**f4**)	Methanol extract	78.9 µg/mL	1.26	[6]
*Pf*	n.d.	*S. repens* Burch. ex Benth. (**f5**)	Essential oil	1.68 µg/mL	5.28	[6]
*Pf*	Chloroquine-resistant *Pf* FCR-3 strain	*S. africana–caerulea* L. (**l**)	Essential oil	4.76 µg/m	n.d.	[45]
*Pf*	Chloroquine-resistant *Pf* FCR-3 strain	*S. africana–lutea* L. (**m**)	Chloroform/MeOH (1:1)	15.86 µg/mL	n.d.	[45]
*Pf*	Chloroquine-resistant *Pf* FCR-3 strain	*S. africana–lutea* L (**m1**)	Essential oil	5.45 µg/mL	n.d.	[45]
*Pf*	Chloroquine-resistant *Pf* FCR-3 strain	*S. albicaulis* Benth. (**n**)	Chloroform/MeOH (1:1)	15.83 µg/mL	n.d.	[45]
*Pf*	Chloroquine-resistant *Pf* FCR-3 strain	*S. albicaulis* Benth. (**n1**)	Essential oil	6.41 µg/mL	n.d.	[45]
*Pf*	Chloroquine-resistant *Pf* FCR-3 strain	*S. muirii* L. Bol. (**o**)	Chloroform/MeOH (1:1)	11.87 µg/mL	n.d.	[45]
*Pf*	Chloroquine-resistant *Pf* FCR-3 strain	*S. muirii* L. Bol. (**o1**)	Essential oil	5.93 µg/mL	n.d.	[45]
*Pf*	Chloroquine-resistant *Pf* FCR-3 strain	*S. lanceolata* Lam. (**p**)	Chloroform/MeOH (1:1)	26.01 µg/mL	n.d.	[45]
*Pf*	Chloroquine-resistant *Pf* FCR-3 strain	*S. lanceolata* Lam. (**p1**)	Essential oil	7.83 µg/mL	n.d.	[45]
*Pf*	Chloroquine-resistant *Pf* FCR-3 strain	*S. garipensis* E. Mey. (**q**)	Chloroform/MeOH (1:1)	13.95 µg/mL	n.d.	[45]
*Pf*	Chloroquine-resistant *Pf* FCR-3 strain	*S. dolomitica* Codd (**r**)	Chloroform/MeOH (1:1)	7.62 µg/mL	n.d.	[45]
*Pf*	Chloroquine-resistant *Pf* FCR-3 strain	*S. dolomitica* Codd (**r1**)	Essential oil	4.81 µg/mL	n.d.	[45]
*Pf*	Chloroquine-resistant *Pf* FCR-3 strain	*S. disermas* L. (**s**)	Chloroform/MeOH (1:1)	24.17 µg/mL	n.d.	[45]
*Pf*	Chloroquine-resistant *Pf* FCR-3 strain	*S. chamelaeagnea* Berg. (**t**)	Chloroform/MeOH (1:1)	8.71 µg/mL	n.d.	[45]
*Pf*	Chloroquine-resistant *Pf* FCR-3 strain	*S. chamelaeagnea* Berg. (**t1**)	Essential oil	8.63 µg/mL	n.d.	[45]
*Pf*	Chloroquine-resistant *Pf* FCR-3 strain	*S. aurita* L.f. (**u**)	Chloroform/MeOH (1:1)	8.92 µg/mL	n.d.	[45]
*Pf*	Chloroquine-resistant *Pf* FCR-3 strain	*S. namaensis* Schinz (**v**)	Chloroform/MeOH (1:1)	25.38 µg/mL	n.d.	[45]
*Pf*	Chloroquine-resistant *Pf* FCR-3 strain	*S. runcinata* L.f. (**v1**)	Chloroform/MeOH (1:1)	16.61 µg/mL	n.d.	[45]
*Pf*	Chloroquine-resistant *Pf* FCR-3 strain	*S. runcinata* L.f. (**v2**)	Essential oil	1.20 µg/mL	n.d.	[45]
*Pf*	n.d.	*S. runcinata* L.f. (**v3**)	MeOH extract	29 µg/mL	>3.44	[6]
*Pf*	n.d.	*S. runcinata* L.f. (**v4**)	Essential oil	1.23 µg/mL	2.06	[6]
*Pf*	Chloroquine-resistant *Pf* FCR-3 strain	*S. schlechteri* Briq. (**w**)	Chloroform/MeOH (1:1)	17.51 µg/mL	n.d.	[45]
*Pf*	Chloroquine-resistant *Pf* FCR-3 strain	*S. stenophylla* Burch. Ex. Benth. (**x1**)	Chloroform/MeOH (1:1)	6.5 µg/mL	n.d.	[45]
*Pf*	Chloroquine-resistant *Pf* FCR-3 strain	*S. stenophylla* Burch. Ex. Benth. (**x2**)	Essential oil	4.13 µg/mL	n.d.	[45]
*Pf*	n.d.	*S. stenophylla* Burch. Ex. Benth. (**x3**)	MeOH-extract	17.03 µg/mL	1.27	[6]
*Pf*	n.d.	*S. stenophylla* Burch. Ex. Benth. (**x4**)	Essential oil	4.38 µg/mL	1.53	[6]
*Pf*	Chloroquine-resistant *Pf* FCR-3 strain	*S. verbenaca* L. (**y**)	Chloroform/MeOH (1:1)	23.97 µg/mL	n.d.	[45]
*Pf*	Chloroquine-resistant *Pf* FCR-3 strain	*S. radula* Benth. (**z1**)	Chloroform/MeOH (1:1)	3.91 µg/mL	n.d.	[45]
*Pf*	Chloroquine-resistant *Pf* FCR-3 strain	*S. radula* Benth. (**z2**)	Essential oil	13.5 µg/mL	n.d.	[45]
*Pf*	Chloroquine resistance Pf K1 strain	*S. chudaei* Batt. & Trab. (**Aa**)	n-Hexane-extract	4.91 ± 2.91 µg/mL	n.d.	[47]
*Pf*	3D7-chloroquine sensitive strain	*S. chudaei* Batt. & Trab. (**Aa**)	n-Hexane extract	5.69 ± 0.3 µg/mL	n.d.	[47]
*Pf*	Chloroquine resistance Pf K1 strain	*S. chudaei* Batt. & Trab. (**Aa1**)	Chloroform-extract	6.41 ± 0.31 µg/mL	n.d.	[47]
*Pf*	3D7-chloroquine sensitive strain	*S. chudaei* Batt. & Trab. (**Aa1**)	Chloroform-extract	5.69 ± 2.71 µg/mL	n.d.	[47]
*Pf*	Chloroquine resistance Pf K1 strain	*S. chudaei* Batt. & Trab. (**Aa2**)	Ethanolic-extract	7.99 ± 1.06 µg/mL	n.d.	[47]
*Pf*	3D7-chloroquine sensitive strain	*S. chudaei* Batt. & Trab. (**Aa2**)	Ethanolic-extract	8.43 ± 0.38 µg/mL	n.d.	[47]
*Pf*	Chloroquine resistance Pf K1 strain	*S. chudaei* Batt. & Trab. (**Aa3**)	Essential oil	2.39 ± 0.24 µg/mL	n.d.	[47]
*Pf*	3D7-chloroquine sensitive strain	*S. chudaei* Batt. & Trab. (**Aa3**)	Essential oil	2.40 ± 0,77 µg/mL	n.d.	[47]
*Pf*	Chloroquine sensitive D6 strain	*S. lavandulifolia* Vahl (**h**)	Essential oil	47% inhibition at 15.86 µg/mL	n.d.	[20]

**Table 8:** Biological activity of extracts of *Salvia* spp. and their isolated compounds against *Plasmodium* (n.d./n.f. = not determined/not found).

Parasite	Strain	Plant Species	IC_50_ Extract	Isolated Compound	Molecular Formula	Molar Mass (g/mol)	IC_50_ of the Compound	SI	Ref.
*Pf*	K1 strain	*S. miltiorrhiza* Bunge.	34% inhib. at 0.81 µg/mL	miltirone (**3**)	C_19_H_22_O_2_	282.38	0.5 µM	2.6	[4]
*Pf*	K1 strain	*S. miltiorrhiza* Bunge.	34% inhib. at 0.81 µg/mL	methylenetanshinquinone (**4**)	C_18_H_14_O_3_	278.09	6.3 µM	1.9	[4]
*Pf*	K1 strain	*S. miltiorrhiza* Bunge.	34% inhib. at 0.81 µg/mL	tanshinone I (**5**)	C_18_H_12_O_3_	276.08	7.2 µM	1.7	[4]
*Pf*	K1 strain	*S. miltiorrhiza* Bunge.	34% inhib. at 0.81 µg/mL	tanshinone IIa (**6**)	C_19_H_18_O_3_	294.13	4 µM	1.9	[4]
*Pf*	K1 strain	*S. miltiorrhiza* Bunge.	34% inhib. at 0.81 µg/mL	methyl-tanshinonate (**7**)	C_20_H_18_O_5_	338.12	4.1 µM	1.7	[4]
*Pf*	K1 strain	*S. miltiorrhiza* Bunge.	34% inhib. at 0.81 µg/mL	11-hydroxymiltiodiol (**8**)	C_19_H_22_O_3_	298.16	≥30 µM	n.d.	[4]
*Pf*	K1 strain	*S. miltiorrhiza* Bunge.	34% inhib. at 0.81 µg/mL	cryptotanshinone (**9**)	C_19_H_20_O_3_	296.14	16.1 µM	0.3	[4]
*Pf*	K1 strain	*S. sahendica* Boiss. & Buhse	n.d.	12-deoxy-salvipisone (**10**)	C_20_H_24_O_2_	296.18	8.8 µM	0.1	[23]
*Pf*	K1 strain	*S. sahendica* Boiss. & Buhse	n.d.	sahandinone (**11**)	C_20_H_24_O_2_	296.18	5.1 µM	0.1	[23]
*Pf*	K1 strain	*S. sahendica* Boiss. & Buhse	n.d.	12-deoxy-6,7-dehydroroyleanone (**12**)	C_20_H_26_O_2_	298.19	17.8 µM	0.6	[23]
*Pf*	K1 strain	*S. sahendica* Boiss. & Buhse	n.d.	7α-acetoxyroyleanone (**13**)	C_22_H_30_O_5_	374.21	1.3 µM	0.1	[23]
*Pf*	K1 strain	*S. sahendica* Boiss. & Buhse	n.d.	Δ^9^-ferruginol (**14**)	C_20_H_28_O	284.21	0.9 µM	18.2	[23]
*Pf*	K1 strain	*S. sahendica* Boiss. & Buhse	n.d.	ferruginol (**15**)	C_20_H_30_O	286.23	0.9 µM	15.6	[23]
*Pf*	K1 strain	*S. sahendica* Boiss. & Buhse	n.d.	sahandol (**16**)	C_20_H_24_O_2_	296.18	4.7 µM	3.3	[23]
*Pf*	K1 strain	*S. sahendica* Boiss. & Buhse	n.d.	sahandone (**17**)	C_21_H_26_O_3_	326.19	17.2 µM	0.7	[23]
*Pf*	K1 strain	*S. hydrangea* DC. ex Benth.	3.2 µg/mL	salvadione C (**22**)	C_30_H_40_O_5_	480.29	1.43 µM	86.2	[25]
*Pf*	K1 strain	*S. hydrangea* DC. ex Benth.	3.2 µg/mL	perovskone B (**23**)	C_30_H_40_O_4_	464.29	18 µM	69.6	[25]
*Pf*	3D7 strain	*S. hydrangea* DC. ex Benth.	<12.5 µg/mL	oleanolic acid (**33**)	C_30_H_48_O_3_	456.36	19.3 µM	n.d.	[8]
*Pf*	K1 strain	*S. miltiorrhiza* Bunge.	34% inhib. at 0.81 µg/mL	1-oxomiltirone (**41**)	C_19_H_20_O_3_	296.14	≥30 µM	n.d.	[4]
*Pf*	K1 strain	*S. miltiorrhiza* Bunge.	34% inhib. at 0.81 µg/mL	1,2-dihydrotanshinquinone (**42**)	C_18_H_14_O_3_	278.09	5.3 µM	0.8	[4]
*Pf*	NF54 strain	*S. austriaca* Jacq.	n.d.	taxodione (**18**)	C_20_H_26_O_3_	314.19	1.9 µM	1.0	[24]
*Pf*	NF54 strain	*S. austriaca* Jacq.	n.d.	15-deoxy-fuerstione (**19**)	C_20_H_26_O_2_	298.19	>167 µM	n.d.	[24]
*Pf*	NF54 strain	*S. austriaca* Jacq.	n.d.	7-(2′-oxohexyl)-taxodion (**20**)	C_26_H_36_O_4_	412.26	3.37 µM	0.9	[24]
*Pf*	NF54 strain	*S. austriaca* Jacq.	n.d.	taxodone (**21**)	C_20_H_28_O_3_	316.20	3.66 µM	1.1	[24]
*Pf*	D6 strain	*S. deserta* Schang.	n.d.	taxodione (**18**)	C_20_H_26_O_3_	314.19	10.49 μM (3.297 mg/L)	>1.4	[39]
*Pf*	W2 strain	*S. deserta* Schang.	n.d.	taxodione (**18**)	C_20_H_26_O_3_	314.19	9.66 μM (3.036 mg/L)	>1.6	[39]
*Pf*	D6 strain	*S. deserta* Schang.	n.d.	ferruginol (**15**)	C_20_H_30_O	286.23	5.64 μM (1.616 mg/L)	0.8	[39]
*Pf*	W2 strain	*S. deserta* Schang.	n.d.	ferruginol (**15**)	C_20_H_30_O	286.23	6.35 μM (1.817 mg/L)	0.7	[39]
*Pf*	D6 strain	*S. deserta* Schang.	n.d.	7-o-acetylhorminone (**30**)	C_22_H_30_O_5_	374.21	>12.72 μM (>4.760 mg/L)	1	[39]
*Pf*	W2 strain	*S. deserta* Schang.	n.d.	7-o-acetylhorminone (**30**)	C_22_H_30_O_5_	374.21	>12.72 μM (>4.760 mg/L)	1	[39]
*Pf*	D6 strain	*S. deserta* Schang.	n.d.	horminone (**31**)	C_20_H_28_O_4_	332.20	>14.33 μM (>4.760 mg/L)	1	[39]
*Pf*	W2 strain	*S. deserta* Schang.	n.d.	horminone (**31**)	C_20_H_28_O_4_	332.20	>14.33 μM (>4.760 mg/L)	1	[39]
*Pf*	FCR3 strain	*S. radula* Benth.	3.91 µg/mL	betulafolientriol oxide (**43**)	C_30_H_52_O_4_	476.39	10.39 μM (4.95 µg/mL)	>20	[48]
*Pf*	K1 strain	*S. hydrangea* DC. ex. Benth.	n.d.	hydrangenone (**44**)	C_30_H_42_O_5_	482.30	1.4 µM	6	[49]
*Pf*	FCR3 strain	*S. radula* Benth.	3.91 µg/mL	salvigenin (**45**)	C_18_H_16_O_6_	328.09	74.98 μM (24.60 µg/mL)	>4	[48]
